# Cuproptosis and Immune-Related Gene Signature Predicts Immunotherapy Response and Prognosis in Lung Adenocarcinoma

**DOI:** 10.3390/life13071583

**Published:** 2023-07-19

**Authors:** Zihao Sun, Xiujing Chen, Xiaoning Huang, Yanfen Wu, Lijuan Shao, Suna Zhou, Zhu Zheng, Yiguang Lin, Size Chen

**Affiliations:** 1 Department of Immuno-Oncology, The First Affiliated Hospital of Guangdong Pharmaceutical University, Guangzhou 510080, China; sunzh158@outlook.com (Z.S.); 2112042020@gdpu.edu.cn (X.C.); 2112142161@gdpu.edu.cn (X.H.); msg941118@163.com (Y.W.); shaoshao12071@163.com (L.S.); 2112142159@gdpu.edu.cn (S.Z.); 2112142160@gdpu.edu.cn (Z.Z.); 2 Guangdong Provincial Engineering Research Center for Esophageal Cancer Precision Therapy, The First Affiliated Hospital of Guangdong Pharmaceutical University, Guangzhou 510080, China; 3 Key Laboratory of Cancer Immunotherapy of Guangdong Higher Education Institutes, Guangzhou 510080, China; 4 Research & Development Division, Guangzhou Anjie Biomedical Technology Co., Ltd., Guangzhou 510535, China

**Keywords:** cuproptosis, lung adenocarcinoma, IRGs, prognosis, tumor microenvironment

## Abstract

Cuproptosis and associated immune-related genes (IRG) have been implicated in tumorigenesis and tumor progression. However, their effects on lung adenocarcinoma (LUAD) have not been elucidated. Therefore, we investigated the impact of cuproptosis-associated IRGs on the immunotherapy response and prognosis of LUAD using a bioinformatical approach and in vitro experiments analyzing clinical samples. Using the cuproptosis-associated IRG signature, we classified LUAD into two subtypes, cluster 1 and cluster 2, and identified three key cuproptosis-associated IRGs, NRAS, TRAV38-2DV8, and SORT1. These three genes were employed to establish a risk model and nomogram, and to classify the LUAD cohort into low- and high-risk subgroups. Biofunctional annotation revealed that cluster 2, remarkably downregulating epigenetic, stemness, and proliferation pathways activity, had a higher overall survival (OS) and immunoinfiltration abundance compared to cluster 1. Real-time quantitative PCR (RT-qPCR) validated the differential expression of these three genes in both subgroups. scRNA-seq demonstrated elevated expression of NRAS and SORT1 in macrophages. Immunity and oncogenic and stromal activation pathways were dramatically enriched in the low-risk subgroup, and patients in this subgroup responded better to immunotherapy. Our data suggest that the cuproptosis-associated IRG signature can be used to effectively predict the immunotherapy response and prognosis in LUAD. Our work provides enlightenment for immunotherapy response assessment, prognosis prediction, and the development of potential prognostic biomarkers for LUAD patients.

## 1. Introduction

Lung adenocarcinoma (LUAD), the primary histological type of lung cancer, is one of the most common malignancies and the leading cause of cancer morbidity and mortality in humans [1]. Nearly 70% of lung cancer patients have locally advanced or metastatic disease at diagnosis [2]. With advances in cancer genomics, a group of genes has been identified as drivers of LUAD, including mutations in the epidermal growth factor receptor (EGFR), c-MET, KRAS, and anaplastic lymphoma kinase (ALK) [3]. In patients with stage IV LUAD who do not have driver gene status changes, if there are no obvious contraindications, the use of immune checkpoint inhibitors (ICIs) is strongly recommended in clinical practice [4]. At the same time, LUAD is generally suitable for ICIs therapy due to its high tumor mutational burden (TMB) and strong immunogenicity [5]. Patients with metastatic lung cancer who are eligible for targeted therapy or immunotherapy live longer, with 5-year survival rates ranging from 15% to 50% [6]. Although a subset of lung cancer patients experience long-term clinical benefit with ICIs, most patients experience disease progression during or after treatment [7]. Immune checkpoint therapy has become an indispensable treatment modality today, and programmed death ligand-1 (PD-L1) and tumor mutational burden have been widely used as biomarkers to assess the suitability of patients for immunotherapy; however, biomarkers of ICIs therapy in NSCLC remain elusive [8,9].

Copper is an important metal element in the human body and is involved in many biological processes, including mitochondrial respiration, iron absorption, antioxidation and detoxification, wound healing, angiogenesis, neurotransmitter synthesis, and regulation of normal cell and tumor growth [10,11,12]. Previous studies have found that copper can promote tumor progression by stimulating endothelial cell proliferation and migration [13,14], promoting angiogenesis [15], and regulating PD-L1 expression [16]. Additionally, copper can induce multiple forms of cell death, including apoptosis and autophagy, through various mechanisms, including reactive oxygen species (ROS) accumulation, proteasome inhibition, and anti-angiogenesis [17]. A novel pattern of cell death, named cuproptosis, was recently reported, and it was elucidated that copper can interact with lipoylated components of the tricarboxylic acid (TCA) cycle in mitochondria, triggering the polymerization of lipid acylated mitochondrial proteins and the loss of iron sulfide (Fe-S) cluster proteins, ultimately leading to proteotoxic stress and cell necrosis [18]. Meanwhile, the authors found that the susceptibility of lung cancer cells to cuproptosis was increased by glutathione depletion, suggesting that cuproptosis is associated with lung cancer.

Several previous studies have shown that certain immune-related genes (IRGs) can serve as prognostic biomarkers in multiple cancers, including LUAD [3,19,20,21]. Unfortunately, few studies have analyzed the combined roles of IRGs and cuproptosis in LUAD classification and prognosis prediction, and current tools to help predict prognosis in LUAD are imprecise [22]. Hence, it is vitally important to reidentify the subtypes of LUAD and filter for genetic signatures with prognostic value. In this paper, we reclassified LUAD into two subtypes based on cuproptosis-associated IRGs, with significant prognostic differences between them. In addition, a predictive model based on three cuproptosis-associated IRGs was constructed and evaluated, with individual risk levels being quantified based on risk scores. We also investigated the patient’s susceptibility to immunotherapy utilizing The Cancer Immunome Atlas (TCIA). In vitro experiments were performed to validate the expression profile of the key genes identified. Our findings demonstrate that the risk model based on cuproptosis-related IRGs can be used to predict prognosis and response to ICIs in LUAD patients.

## 2. Materials and Methods

### 2.1. Data Source and Preprocessing

The data, mainly derived from The Cancer Genome Atlas (TCGA), the Immunology Database and Analysis Portal (ImmPort) [23], and The Cancer Imaging Archive (TCIA) [24], were used for integrative analysis. The gene expression data and clinical information of LUAD patients were downloaded from TCGA. Transcriptome data of 19 cuproptosis-associated genes were obtained from the TCGA-LUAD dataset [18]. A list of IRGs (n = 2848) was obtained from ImmPort. Sensitivity data for immunotherapy corresponding to the TCGA-LUAD cohort were extracted from TCIA. Single-cell RNA-seq (scRNA-seq) data were derived from the GSE203360, GSE149655, and GSE131907 of the Gene Expression Omnibus (GEO). And scRNA-seq profiles of one malignant pleural effusions sample were obtained from The First Affiliated Hospital of Guangdong Pharmaceutical University (TFAHGPU).

### 2.2. Unsupervised Clustering for Cuproptosis-Related IRGs

First, IRGs and cuproptosis-related gene expression matrices were extracted from the TCGA-LUAD gene expression matrix of 516 tumor samples, and 64 cuproptosis-related IRGs were screened by coexpression analysis (Supplementary Materials Table S1). Then, univariate Cox regression analysis was applied to filter 17 cuproptosis-related IRGs that were significantly associated with LUAD prognosis (*p* < 0.05). Moreover, consensus classification of the 17 cuproptosis-related IRGs was conducted by using the R package “ConsensusClusterPlus” [25], and the parameters clusterAlg, distance, reps, pltem, and pFeature were set to pam, euclidean, 100, 0.8, and 1, respectively. Next, according to the optimal cluster number, survival analysis was employed to clarify the survival differences between the two clusters. Finally, based on the clustering results, data downscaling was performed using principal component analysis (PCA), principal coordinate analysis (PCoA), and t-distributed stochastic neighbor embedding (tSNE).

### 2.3. Cell Infiltration and Biological Characteristics of Both Subtypes

Gene set variation analysis (GSVA) was performed on two subtypes using “c2.cp.kegg.v7.5.1.symbols.gmt” derived from MSigDB and a list of formerly published and biologically concerned gene signatures as the annotated gene set (Supplementary Materials Table S2). Single-sample gene set enrichment analysis (ssGSEA) was accessed to infer the abundance of 28 immunoinfiltrating cells in the TCGA-LUAD cohort [26]. Moreover, five other algorithms, namely, MCP-counter [27], xCell [28], TIMER2.0 (https://cistrome.shinyapps.io/timer/, accessed on 1 May 2023), CIBERSORT [29], and ESTIMATE [30], were implemented to examine the accuracy and robustness of the ssGSEA results.

### 2.4. Derivation of the Cuproptosis Prognostic Signature

First, the clinical data of 476 cases in the TCGA-LUAD cohort and the expression matrix of cuproptosis-related IRGs were merged by sample name and split into a training cohort (70%) and testing cohort (30%). The cuproptosis-associated IRGs were then filtered by univariate Cox analysis with *p*-values less than 0.05 in the training set. The genes required for modeling were further selected by least absolute shrinkage and selection operator (LASSO) regression analysis using the R package “glmnet” [31]. Stepwise regression was employed to construct an optimal multivariate Cox regression model for the cuproptosis-related IRGs screened by LASSO, and then the risk scores of each sample in the training and testing sets were calculated as follows:

(1) $$\mathrm{Risk}\;\mathrm{score}=\overset{n}{\underset{i=1}{\sum }}\exp_{i}{\times \;\beta }_{i}\;$$

*β* and *exp* represent the regression coefficient and expression of each cuproptosis-associated IRG, respectively. *i* represents the number of each gene. To prevent bias and guarantee the robustness of the model, the testing set and whole cohort were divided into low- and high-risk groups based on the median risk scores of the training set.

### 2.5. Real-Time Quantitative PCR (RT-qPCR)

The tumor specimens used in this study were approved (Approval No. 69, 2022) by the Human Ethics Committee of the First Affiliated Hospital of Guangdong Pharmaceutical University. Lung cancer specimens and associated clinical data including survival data from 12 patients with LUAD were collected for the study. RT-qPCR was performed to examine the expression levels of three key genes in these 12 samples. Primer sequences of NRAS (F GTGGAGCTTGAGGTTCTTGC; R CTGGATTGTCAGTGCGCTTT), TRAV38-2DV8 (F CCTGTCTTGAATTTAGCATGGCTC; R GCGAATAACGAGAATCATCTGCC), and SORT1 (F GACCTTGGGGCTCTGGAATTATG; R CCCTTGATCTGTTGAAACGTGGA) were designed based on the gene sequences on NCBI and detected by RT-qPCR using cDNA as a template. NAnodrop2000c was used to detect the RNA concentration in the samples. The samples were heated at 70 °C for 5 min to disrupt the RNA secondary structure. The reagents were then sequentially added using the First Strand cDNA Synthesis Kit, and the samples were kept at 50 °C for 30 min, followed by heating at 85 °C for 5 min and cooling on ice to prepare the cDNA. The qPCR was performed using the Ipure SYBR Green qPCR Master Mix kit, and the gene expression levels were normalized to GAPDH levels. The prognostic risk score of patients was then calculated based on the results of RT-qPCR using Equation (1), and the median value of the risk score of the training set was applied to split the 12 patients into low- and high-risk groups.

### 2.6. Establishment and Assessment of the Nomogram

We first executed a univariate Cox regression analysis on risk scores and clinical signatures in the TCGA-LUAD cohort, followed by a multivariate Cox regression analysis. The nomogram was then plotted by the “regplot” R package [32]. The area under the curve (AUC) and C-index were computed by the R package “timeROC” to assess whether the predicted values of the model were aligned with the actual values [33]. The calibration and decision curves were plotted with the R packages “rms” and “ggDCA”, respectively.

### 2.7. Single-Cell RNA Sequencing (scRNA-Seq) Data Analysis

Quality control of the 10× scRNA-seq data was executed using the R package “Seurat” [34]. The low-quality cells were filtered according to the following conditions: (1) total UMI counts >1000; (2) mitochondrial gene expression <20%; (3) gene numbers >500; and (4) erythrocyte gene expression <3%. Single-cell data integration was performed using the Harmony algorithm. We used NormalizeData, FindVariableFeatures with nfeatures = 2000, ScaleData, RunPCA, and FindNeighbors with the first 16 PCs and FindClusters with resolution = 0.5 to further process the data, and all other parameters remained the same. We combined references and known classical markers to label the obtained cell clusters as conventional dendritic cells (cDCs) (CD207, CD1A, CD1C, FCER1A); plasmacytoid DCs (pDCs) (CLEC4C, LILRB4, NRP1); T cells (TRAC, CD3G, CD3E, CD3D) [35]; B cells (CD19, CD79A, MS4A1) [36]; monocytes (VCAN, CD14, CD36); macrophages (CD163, MSR1, C1QA, FCGR3A, CD68, CCL18, CXCL10); endothelial cells (EGFL7, PECAM1); epithelial cells (CDH1, KRT18, KRT19, EPCAM); fibroblasts (COL1A2, COL1A1, DCN, THY1, FGF7); plasma cells (MZB1, JCHAIN, SDC1, XBP1); neutrophils (FCGR3B, CXCR2, CSF3R, G0S2) [37]; and mast cells (KIT, GATA2, TPSAB1, CPA3, MS4A2, TPSB2) [36,38]. In addition, we used SingleR for cell subpopulations annotation [39]. The nuclear density maps of marker genes expression in each cell type were developed by “Nebulosa” software (Version 1.4.0).

### 2.8. Biological Features Analyses

To further identify the prognostic differences between the two subgroups, we conducted gene set enrichment analysis (GSEA) [40] on the samples using “c2.cp.kegg.v7.5.1.symbols.gmt” and gene sets compiled from references (Supplementary Materials Table S3) as the annotated gene sets. The statistically significant *p*-value was less than 0.05. Gene Ontology (GO) analysis was conducted using the “clusterProfile” software. (Version 4.2.2)

### 2.9. Gene Mutation and Immunotherapy Response Analysis

Mutation data of LUAD were extracted from the official TCGA website, and TMB was calculated separately for the two subgroups. The “maftools” [41] software (version 2.10.05) was employed to display the top 10 gene mutation maps for tow subgroups. Furthermore, we assessed statistical significance in immune checkpoint-related genes’ expression and immunotherapy sensitivity in both subgroups.

### 2.10. Statistical Analysis

In this study, the Wilcoxon test was performed to analyze statistical differences between the two groups that were not normally distributed. Correlation between two variables with non-normal distribution was assessed by Spearman’s rank correlation test. The results of the RT-qPCR were compared between two groups using an unpaired t-test. Difference in survival curves was evaluated by a log-rank test. The value 0.05 was determined to be the significance threshold for *p*-value. Data analysis and visualization of the results were implemented with R 4.1.3 software.

## 3. Results

### 3.1. Confirmation of Novel Subtypes of Cuproptosis-Associated IRGs in LUAD

**Figure 1:**
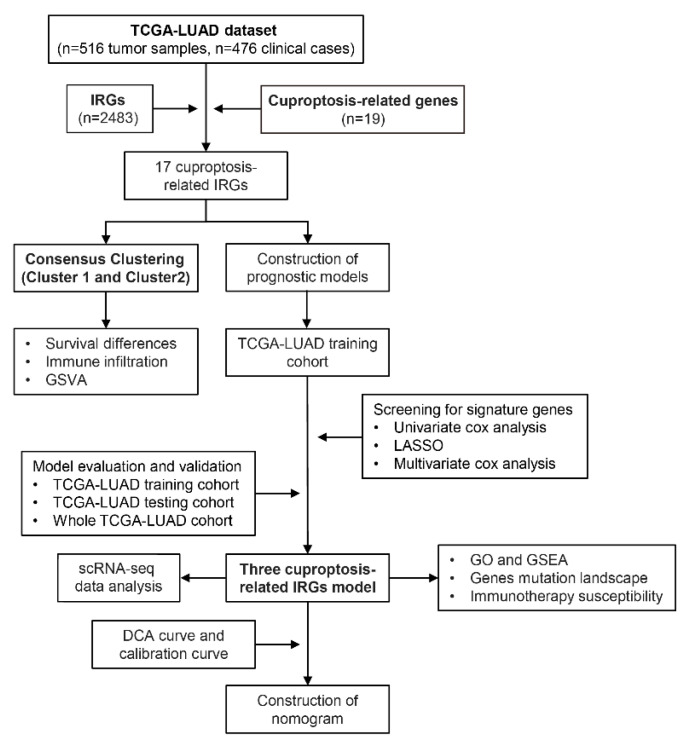
The flowchart of our study. A total of 516 lung adenocarcinoma (LUAD) samples from TCGA were included, and cuproptosis-related IRGs were identified. Tumor samples from TCGA-LUAD were then classified into two subtypes. Clinical prognostic features were constructed based on cuproptosis-related IRGs, and immunotherapy sensitivity analysis was performed.

**Figure 2:**
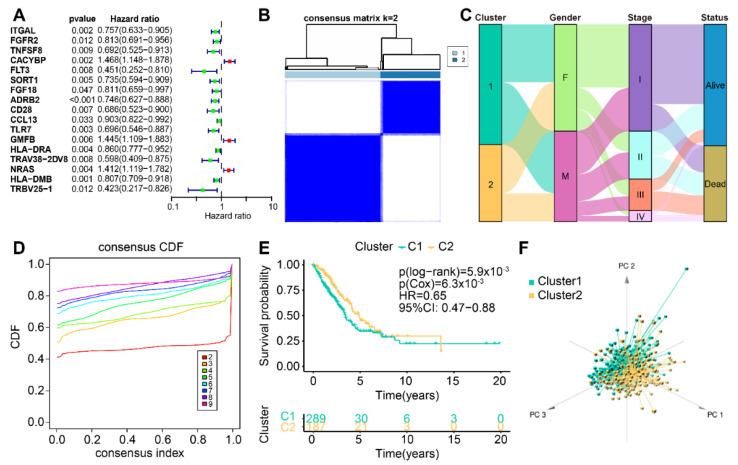
Gene coexpression and consensus clustering. (**A**) Forest plot of 17 cuproptosis-related IRGs. (**B**) Consensus matrix heatmap of TCGA-LUAD samples with k = 2. (**C**) Alluvial diagram showing changes in two clusters and clinical features. (**D**) CDF plot showing the consensus distribution for each cluster. (**E**) Overall survival curves of the two clusters. (**F**) Principal component analysis of 17 cuproptosis-related IRGs classified the cohort into two subtypes.

The flow chart of our study is shown in Figure 1. Through coexpression analysis of cuproptosis-associated genes and IRGs, 64 cuproptosis-associated IRGs were filtered. Then, 17 cuproptosis-associated IRGs with prognostic value were screened by one-way Cox regression analysis in 476 patients with LUAD (Figure 2A). To explore the classification of cuproptosis subtypes in TCGA-LUAD, an expression matrix of 17 cuproptosis-related IRGs was analyzed using unsupervised clustering. A total of 9 clusters were applied to consensus clustering analysis (Figure 2B and Figure S1A–G), and the optimal number of clusters identified by the delta area plot and cumulative distribution plot of consensus scores was 2 (Figure 2D and Figure S1H). We further analyzed the distribution of clinical features of LUAD patients in the new LUAD classification (Figure 2C). To better elucidate the clinical significance of the LUAD classification, we split the TCGA-LUAD cohort into two groups according to the LUAD classification for survival analysis, and the results indicated that cluster 1 had a worse prognosis compared with cluster 2. (Figure 2E). Meanwhile, PCA was performed on the TCGA-LUAD expression matrix, and the results further confirmed the significant differences between the two subtypes (Figure 2F), which was in accordance with the results of PCoA (Figure S1I) and tSNE (Figure S1J) analyses. Additionally, we found that 6 out of 17 cuproptosis-related IRGs were differentially expressed in the subtypes (Figure S1K).

### 3.2. Differences in TME of Cuproptosis-Associated Subtypes

**Figure 3:**
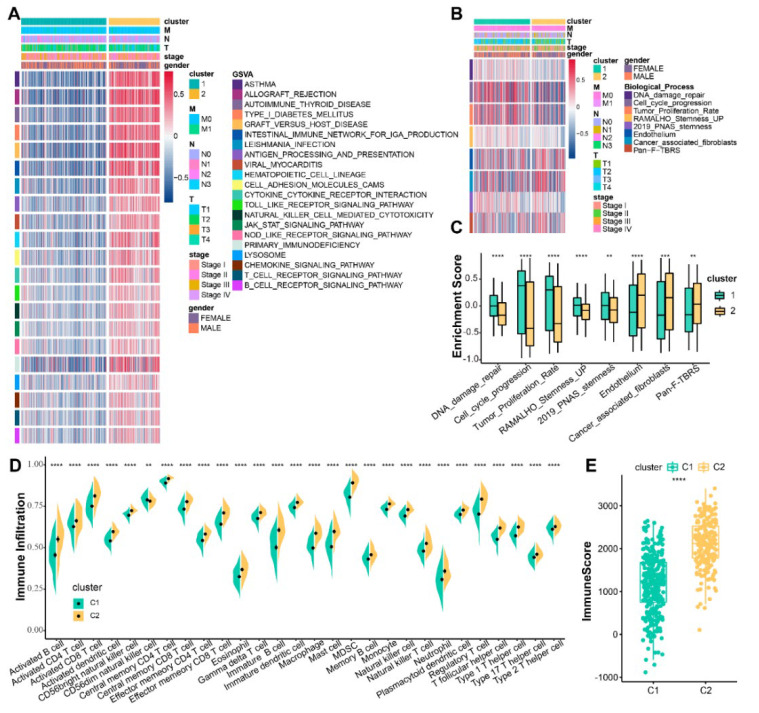
Biological signatures and immune landscape of the two clusters. (**A**) GSVA performed on both subtypes with an annotated gene set from MSigDB. (**B**) Heatmap showing enrichment of signatures in both subtypes. (**C**) Boxplot showing quantification of enrichment of indicated signatures among two subtypes. (**D**) The abundance of 28 tumor-infiltrating lymphocytes in both subtypes was calculated by ssGSEA. The dot denotes the median value. Error bar indicates confidence interval. (**E**) The ESTIMATE calculated the immune scores between the two subtypes. “**”: *p* < 0.01; “***”: *p* < 0.001; “****”: *p* < 0.0001.

To better understand the survival differences in both subtypes, we next investigated the differences in the biological signatures and tumor microenvironment (TME) of the two clusters. The GSVA algorithm performed on the two subtypes demonstrated that immune activation-associated pathways activity was upregulated in cluster 2, such as antigen processing and presentation, T-cell and B-cell receptor-signaling pathways, and natural killer cell-mediated cytotoxicity (Figure 3A). At the same time, pathways activity of tumor-associated biological processes, such as DNA damage repair, proliferation (cell cycle progression and tumor proliferation rate), and stemness (RAMALHO stemness UP and 2019 PNAS stemness), were upregulated in cluster 1, while cluster 2 had a significantly higher enrichment score of interstitial activation pathways (endothelium, cancer-associated fibroblasts, and pan-fibroblast TGF-β response signature (pan-F-TBRS)) than cluster 1 (Figure 3B,C). ssGSEA was performed on two clusters to elucidate the characteristics of TME. The two subtypes demonstrated significant differences in the abundance of immunoinfiltrating cells, with cluster 2 having a dramatically higher infiltration abundance than cluster 1 (Figure 3D and Figure S2A). The 17 cuproptosis-related IRGs were also significantly associated with the abundance of these 28 immunoinfiltrating cells (Figure S2B). To ensure that the results of the ssGSEA were not biased and to demonstrate that the above results are robust and accurate, five other algorithms, namely, ESTIMATE, TIMER2.0, MCP-counter, CIBERSORT, and xCell, were used (Figure 3E and Figure S2C–G).

### 3.3. Creation of the Cuproptosis-Associated Prognostic Signature

**Figure 4:**
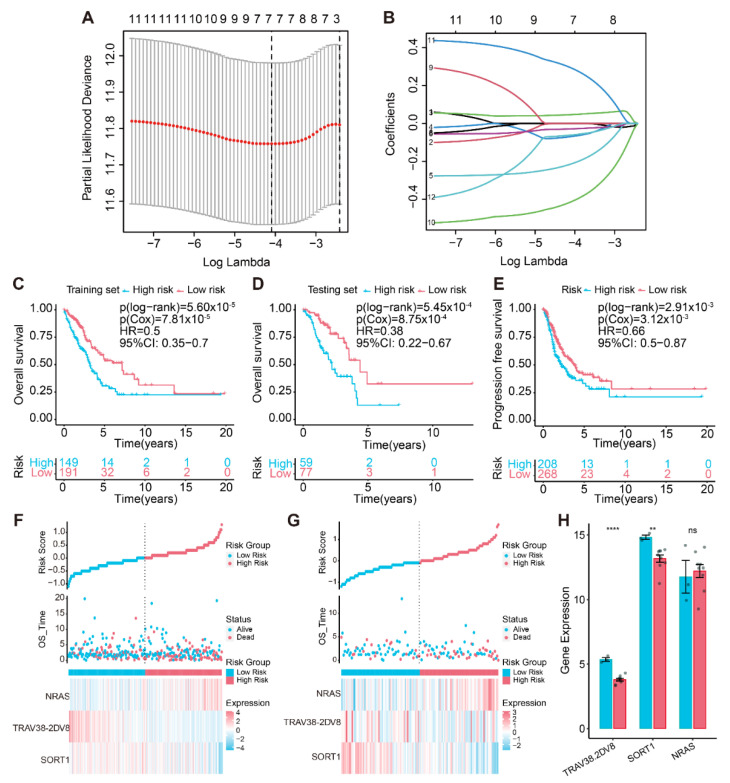
Building of a risk model using three IRGs associated with cuproptosis. (**A**) The LASSO analysis selected seven features by optimal λ. (**B**) Distribution of the coefficients and log (λ) of the LASSO regression. Survival curves of two risk subgroups in the (**C**) training cohort and (**D**) testing cohort. (**E**) Kaplan–Meier curves of progression-free survival for two subgroups in the whole cohort. The risk factor linkage plots in the (**F**) training cohort and (**G**) testing cohort demonstrate the status of patient survival and expression levels of signature genes in response to risk scores. (**H**) Histogram of SORT1, NRAS, and TRAV38-2DV expression levels detected by real-time quantitative PCR (RT-qPCR). “ns”: no statistical significance; “**”: *p* < 0.01; “****”: *p* < 0.0001.

First, to obtain cuproptosis-associated signatures that could be employed to predict LUAD prognosis, 70% of TCGA-LUAD patients served as a training cohort and 30% as a test cohort (Table 1). Univariate Cox regression analysis and LASSO analysis were conducted to identify seven key genes in the training cohort (Figure 4A,B). Then, the best prognostic risk model was screened using stepwise regression, and three prognostic genes were selected, including NRAS, T-cell receptor alpha variable 38-2/delta variable 8 (TRAV38-2DV8), and SORT1. A prognostic risk model for LUAD was developed using these three critical genes, and cuproptosis-related risk scores were calculated using the following equation:

Risk score = −(0.30914682 × Exp SORT1) − (0.68345148 × Exp TRAV38-2DV8) + (0.40691588 × Exp NRAS).(2)

The median risk score (cutoff value: 1.00009) was applied to classify the training cohort, the test cohort, and the whole cohort into two groups.

In the TCGA-LUAD training cohort, the probability of OS was remarkably higher in the low-risk subgroup, and the prognosis was clearly worse for the high-risk subgroup (Figure 4C,F). Likewise, the same results were found in the testing set (Figure 4D,G). In the entire TCGA-LUAD cohort, progression-free survival was compared in both subgroups, demonstrating superior performance in the low-risk subgroup (Figure 4E). The risk scores also accurately predicted the probability of OS for the entire cohort (Figure S3A,B). Furthermore, we performed the Spearman’s test to examine whether the three prognostic genes’ expression levels had a strong relevance to the risk score. The findings revealed that TRAV38-2DV8 and SORT1 had a negative correlation, while NRAS expression level was increased with rising risk scores (Figure S3C–E).

Furthermore, we discovered that patients with different clinical stages and genders had considerably different risk scores (Figure S3F–G). The stratified analyses illustrated that three cuproptosis-associated IRGs’ signatures, regardless of clinical stage and gender, were utilized to reliably detect prognostic differences for patients in the high-risk subgroup (Figure S3H–K). This demonstrated that the cuproptosis-related signature distinguished prognostic differences based on clinical characteristics. The results of the multivariate Cox regression analyses supported those of the univariate Cox regression analyses in that the prognostic influence of age, gender, stage, and TMB in patients declined risk stratification. However, risk stratification resulted in fluctuating prognostic implications for risk scores, indicating that risk stratification was linked to the prognostic value of this feature (Table S4). We performed RT-qPCR on tumor samples from 12 LUAD patients, and the results suggested significant differences in the expression of TRAV38-2DV8 and SORT1 in the low- and high-risk groups and were consistent with the trend in the TCGA cohort, while NRAS expression was not significantly different in the two risk subgroups (Figure 4H). The possible reason for this biased result was the small sample size.

### 3.4. Gene Expression Pattern Analysis

**Figure 5:**
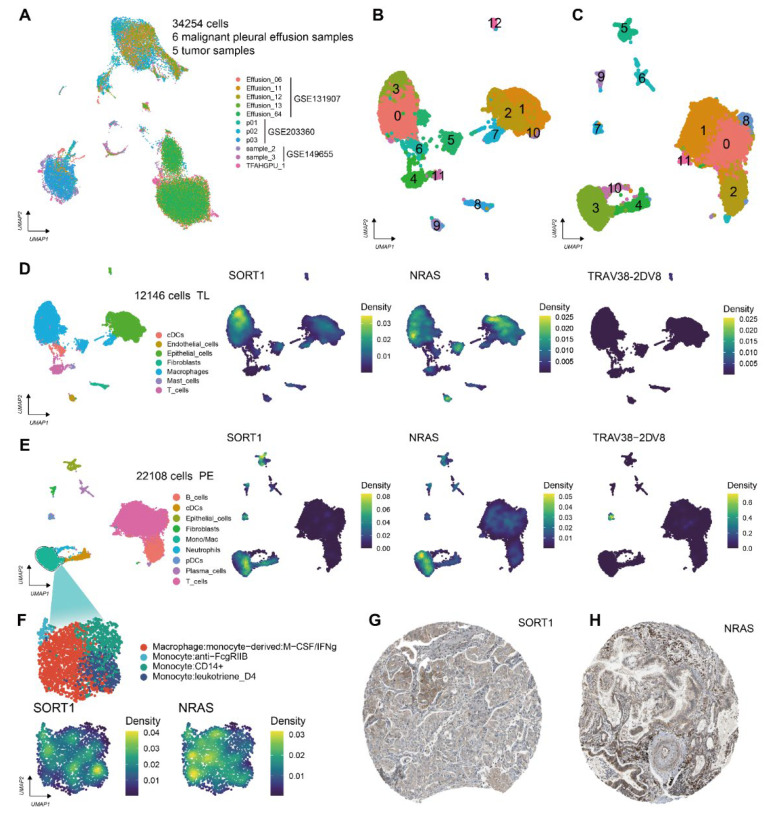
Prognostic gene expression patterns. (**A**) Distribution of the 11 sample cells integrated together. UMAP visualization of the cellular annotation results for (**B**) lung adenocarcinoma (LUAD) tissue (the numbers 0–12 represent the cluster number) and (**C**) malignant pleural effusions (the numbers 0–11 represent the cluster number). Results of cell-type annotation in (**D**) LUAD tissue samples and (**E**) malignant pleural effusion samples and nuclear density maps of SORT1, NRAS, and TRAV38-2DV expression in these cell populations. TL: lung adenocarcinoma tissue samples; PE: malignant pleural effusion samples. (**F**) UMAP visualization of subtype annotation results of Mono/Mac cell cluster in malignant pleural effusion samples and nuclear density maps of SORT1 and NRAS expression. Immunohistochemical atlas of (**G**) SORT1 and (**H**) NRAS.

The result of the single-cell data integration is shown in Figure 5A. We performed dimension reduction and clustering in lung adenocarcinoma tissue samples (Figure 5B) and malignant pleural effusion samples (Figure 5C), respectively, and then manually annotated the cell clusters of these two groups of samples according to marker genes. Cell types of lung adenocarcinoma tissue samples include immune cells (DCs, macrophages, mast cells, and T cells), endothelial cells, epithelial cells, and fibroblasts; B, DCs, epithelial cells, fibroblasts, Mono/Mac, neutrophils, plasma cells, and T cells are present in malignant pleural effusion samples (Figure S4). The expression of SORT1 and NRAS was also observed to be higher in macrophages; however, only pDCs from samples of malignant pleural effusion showed considerable expression of TRAV38-2DV8 (Figure 5D–F). Immunohistochemistry from The Human Protein Atlas (THPA) also confirmed that NRAS and SORT1 expression levels were upregulated in LUAD tissues (Figure 5G,H).

### 3.5. Establishment of a Cuproptosis-Associated IRG Prognostic Risk Model

**Figure 6:**
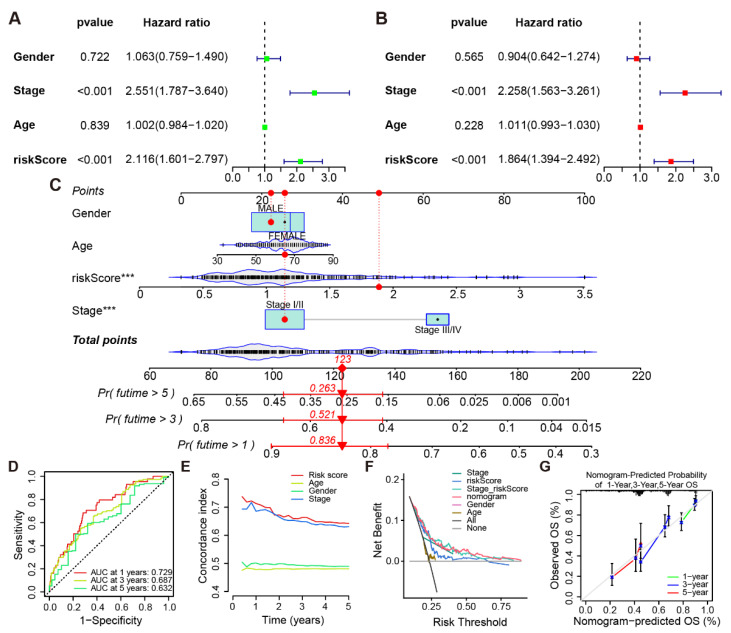
Establishment and performance evaluation of model. (**A**) Univariate and (**B**) multivariate Cox regression analysis of clinical features and risk scores. (**C**) Nomogram including of risk scores and clinical characteristics. (**D**) ROC curves for the precision of the model in predicting patient prognosis. (**E**) C-index curve based on the risk model. (**F**) Decision analysis of clinical features and risk scores to predict clinical benefit in LUAD patients. (**G**) Evaluation of nomogram’s 1-, 3-, and 5-year forecast accuracy. “***”: *p* < 0.001.

Univariate and multivariate Cox regression analyses of clinical characteristics and the risk score of LUAD patients (Figure 6A,B) indicated that risk score had an independent influence on patient prognosis. A nomogram was set up using all the above features (Figure 6C). The nomogram shows the predicted probability of survival for patient number 10. The total score was determined based on the score for each item calculated using the nomogram. The AUC, which were, respectively, 0.729, 0.687, and 0.632 on the ROC curve, measured how well the model predicted the OS probability of LUAD patients at 1, 3, and 5 years (Figure 6D). The C-index of all features in the model for 5-year prognosis prediction of LUAD patients was calculated separately, indicating that the risk score had sufficient predictive power for the patients’ prognosis (Figure 6E). Decision curve analysis (DCA) demonstrated that the nomogram exhibited excellent predictive power with high clinical benefit (Figure 6F). The calibration curves confirmed that the model was highly accurate in projecting the odds for LUAD patients’ 1-, 3-, and 5-year OS (Figure 6G).

### 3.6. Biological Characteristics Analysis of the Prognostic Model

**Figure 7:**
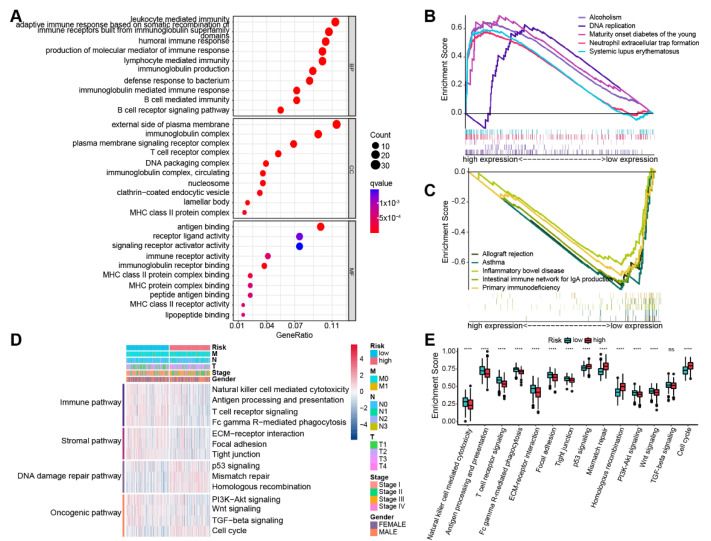
Analysis of biological characteristics. (**A**) GO analysis results of two subgroups. BP: biological process; CC: cellular component; MF: molecular function. GSEA results reveal the top 5 (**B**) upregulated and (**C**) downregulated pathways in both subgroups. (**D**) Enrichment levels of 14 pathways in the two subgroups. (**E**) Differences in enrichment scores for 14 pathways in the two subgroups. “ns”: not significant; “****”: *p* < 0.0001.

Genes that had differential expression between the two subgroups were capitalized on for GO enrichment analysis, and the top 10 terms were retrieved for visualization (Figure 7A). A powerful connection between immune activation and prognostic risk models are suggested by the fact that GO terms were primarily enriched in molecular mechanisms of immune activation, such as pathways related to leukocyte, B cell, and lymphocyte-mediated immunity; MHC class II protein complexes; and pathways related to receptor activity. DNA replication, folate biosynthesis, and systemic lupus erythematosus (SLE) were among the pathways that were upregulated in the high-risk subgroup according to GSEA results for the KEGG pathway (Figure 7B), whereas immune enterocolitis, asthma, allograft rejection, and immunodeficiency-related pathways were dramatically downregulated. (Figure 7C). Furthermore, we performed GSEA using 14 pathways selected from pathway databases and publications and indicated that the high-risk subgroup presented elevated activities of DNA damage repair and cell cycle pathways, while the low-risk subgroup highly expressed immune, stromal, and partial carcinogenesis-related pathways (Figure 7D,E). We further investigated the relevance between enrichment levels of these 14 pathways and risk scores, and the results confirmed this finding, which largely illuminated the reason for the worse prognosis of patients in the high-risk subgroup (Figure S5A,B).

### 3.7. Gene Mutation Landscape and Immunotherapy Susceptibility

**Figure 8:**
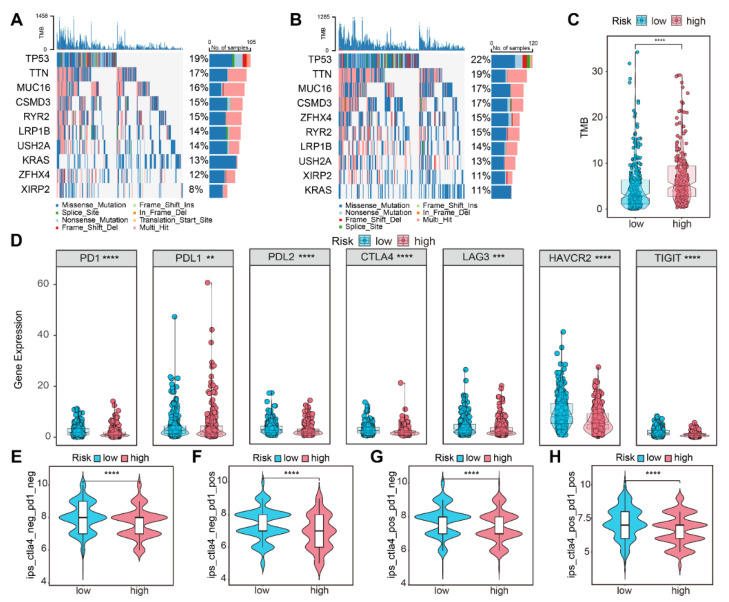
Gene mutation landscape and immunotherapy susceptibility. Mutation profile of the top 10 genes in the (**A**) low- and (**B**) high-risk group. (**C**) Mutational burden in the two subgroups. (**D**) PD-1, PD-L1, PD-L2, CTLA-4, LAG3, HAVCR2, and TIGIT expression differences in the two subgroups. Immune therapy scores in the two subgroups: (**E**) no immunotherapy, (**F**) anti-PD-1therapy, (**G**) anti-CTLA-4 therapy, (**H**) anti-PD-1, and anti-CTLA-4 therapy. “ns”: no statistical significance; “**”: *p* < 0.01; “***”: *p* < 0.001; “****”: *p* < 0.0001.

We analyzed the difference in gene mutation distribution between the two subgroups in the TCGA-LUAD cohort, which demonstrated that tumor mutation burden (TMB) was higher in the high-risk population (Figure 8A–C). In addition, our study found that immune checkpoint-related genes had higher expression in the low-risk population (Figure 8D). Lower clinical efficacy scores for ICIs indicated that patients are less sensitive to immunotherapy. Comparison of efficacy scores in two subgroups of the four treatment groups, either with anti-PD-1 and anti-CTLA4 alone or in combination, revealed higher immunotherapy scores in the low-risk subgroup, indicating that this subgroup was better suited for immunotherapy (Figure 8E–H).

## 4. Discussion

The majority of patients who die from malignancies are patients with lung cancer, and LUAD has the highest proportion of lung cancer occurrence [42]. Analysis of exon copy number profiling, mutation, rearrangements, and DNA methylation in LUAD samples, as well as further assessment of mRNA, miRNA, and protein expression, have demonstrated that LUAD lesions are dramatically heterogeneous [43,44]. Although deep learning and machine learning have been extensively employed for tumor classification and prognostic assessment in recent years [45], markers with accuracy of predicting patient prognosis are still limited. Therefore, an accurate identification of the molecular subtypes of LUAD is crucial for guiding antitumor therapy.

The process of tumor proliferation, invasiveness, metastasis, and migration is strongly linked to TME, and immunoinfiltrating cells play an obviously crucial role in tumor escape and antitumor therapy. It has been ascertained that both innate and adaptive immune cells are present in TME [46]. TME is closely associated with lung cancer heterogeneity and has a major impact on lung carcinogenesis and development. Its dynamic changes depend on infiltrating lymphocytes, cellular regulatory factors, immune-related genes, and protein expression profiles [47]. Todd Golub’s team first proposed the concept and mechanism of cuproptosis, and many studies have found that cuproptosis-related genes are inextricably interconnected with different types of immune cell infiltration, such as LIAS and FDX1 [48,49]. LIPT1 is one of the genes associated with cuproptosis, and Lv H et al. found that in the TME of melanoma patients, the abundance of resting CD4 memory T cells had a positive correlation with the expression of this gene, whereas the abundance of effector T cells and natural killer (NK) cells was inversely correlated. [50]. Furthermore, cuproptosis also has a crucial influence on tumors in other ways, such as increasing glycolysis by downregulating PDHA1 expression and thus promoting gastric cancer development [51].

IRGs are closely bound up with the prognostic evaluation and treatment of tumors. Currently, multiple immune-associated characteristics are applied to identify different subgroups of prognostic patients with LUAD and to predict their prognosis. [3,52,53,54]. Ma KY et al. explored the heterogeneity of IRGs expression in tumors using scRNA-seq data and demonstrated that it has paramount implications for immunotherapy efficacy [55]. However, the impact of cuproptosis-associated IRGs on oncogenesis, invasion and metastasis, prognosis, and treatment of LUAD is currently unclear. Therefore, our study attempted to investigate the comprehensive effects of cuproptosis-associated IRGs in LUAD. We also designated specific cuproptosis-associated IRGs that could be employed to identify subtypes and estimate the prognosis of LUAD.

In this study, we first screened for cuproptosis-related IRGs and separated the TCGA-LUAD cohort into two clusters by consensus clustering. Survival analysis elucidated a considerable difference in prognosis in both subtypes, with the cluster 2 cohort enjoying a lopsided survival advantage. To explore the reasons for the difference, GSVA enrichment analysis found that immune activation-related pathways were upregulated in cluster 2, such as NK cell-mediated cytotoxicity, antigen processing and presentation, T- and B-cell receptor signaling, and cell adhesion molecules. At the same time, various algorithms were conducted to estimate the immune composition of LUAD. The results illuminated that NK cells, CD8+ T cells, natural killer T (NKT) cells, M1 macrophages, and γδ T cells were more abundantly infiltrating in cluster 2. Studies have shown that M1 macrophages, NKT cells, NK cells, and γδ T cells contribute to the inhibition of tumor development [56,57,58]. We also found that compared with cluster 1, 6 of 17 cuproptosis-related IRGs used to construct cuproptosis subtypes, namely, CCL13, TLR7, HLA-DRA, TRAV38-2DV8, HLA-DMB, and TRBV25-1, were upregulated in cluster 2. Zhao W et al. found that CCL13 can be utilized to indicate the intratumoral heterogeneity of immunoinfiltration in lung carcinoma and its association with OS [59]. TLR7 stimulation leads to decreased expression of CD200R in immunoinfiltrating cells (CD45+), resulting in anti-tumor effects in multiple tumors [60,61]. Jie Mei et al. found that human leukocyte antigen-DR alpha (HLA-DRA) expression levels were reduced in NSCLC tissues, related to TME inflammation, and predictive of the response of NSCLC to ICIs. This study also found that HLA-DRA was expressed in tumor and immune cells [62]. Fling SP et al. showed that HLA-DMB, encoding a component required for the assembly of MHC class II intramolecular peptides, is a gene that functionally maps between HLA-DP and HLA-DQ, and is involved in class II antigen presentation [63].

Next, Cox regression analysis and LASSO analysis were executed to screen for three cuproptosis-associated IRGs (NRAS, TRAV38-2DV8, and SORT1) and to construct a prognostic risk model. Validation with internal and external data ascertained that the model can precisely distinguish risk subgroups of LUAD patients and provide a clinical reference to facilitate quantitative risk management of LUAD patients. In addition, we performed RT-qPCR using tumor samples from 12 patients and initially verified that TRAV38-2DV8 and SORT1 expression was remarkably elevated in the low-risk group. Due to the small sample size, NRAS expression had no significant differences in the two subgroups. According to a recent study, NRAS has a responsibility in immune cell infiltration in the TME and has an independent effect on the prognosis of LUAD patients. Its expression is strongly inversely correlated to the prognosis of LUAD [64]. Mutated or overexpressed NRAS promotes tumor lung colonization by regulating the expression of IL-8-related chemokines and initiating interactions between tumor cells, pulmonary blood vessels, and myeloid cells [65]. Several studies have demonstrated that SORT1 expression is elevated in several types of tumors, which is correlated with a poor prognosis, such as liver [66], gastric [67], prostate [68], and colorectal cancers [69]. We demonstrated that the expression levels of SORT1 and NRAS were obviously elevated in macrophages in LUAD samples by scRNA-seq data analysis. To date, the prominence of TRAV38-2DV8 and SORT1 for prognostic prediction and clinical therapeutic efficacy assessment of LUAD have not yet been thoroughly investigated in relevant studies, and more research is required to evaluate the effect of SORT1 and NRAS expression levels in macrophages on the occurrence and progression of LUAD. Nomograms can calculate event probabilities based on features in prognostic models to assess patient prognostic risk levels and are widely applied in prognostic prediction in cancer [70]. In this study, a nomogram was established to predict OS of patients at 1, 3, and 5 years. The calibration curve and the DCA curve revealed that the nomogram has a great clinical predictive value.

Moreover, GO analysis and GSEA were carried out in two subgroups in order to clarify the molecular mechanisms of the prognostic model. Cell cycle and DNA damage-repair-related pathways were strongly expressed in the high-risk subgroup with a poor prognosis, whereas immunological-, stromal-, and certain oncogenic-associated pathways were highly expressed in the low-risk subgroup with improved prognosis. We also found that the low-risk subgroup was more responsive to immunotherapy and had a lower frequency of mutations. This largely explained why the low-risk subgroup had a better prognosis.

This research has several restrictions. First, the statistical study used data from open databases, and more research is required to determine the function of cuproptosis-associated IRGs, including NRAS, TRAV38-2DV8, and SORT1, in the TME of LUAD. Furthermore, we have not yet assessed the risk model using a clinical cohort due to the tiny clinical sample that was gathered.

## 5. Conclusions

In conclusion, using the cuproptosis-associated IRG signature, we identified three key cuproptosis-associated IRGs, NRAS, TRAV38-2DV8, and SORT1, and meaningfully categorized LUAD into two subgroups with prominent differences in prognosis, molecular features, and immunoinfiltration abundance. More importantly, we successfully established a prognostic prediction model based on the three IRGs connected to cuproptosis, and the model has superior performance in predicting the immunotherapy response and prognosis of LUAD. These three key genes expression profile in clinical samples from patients with LUAD is validated. Furthermore, we elucidated how cuproptosis-associated IRGs functioned through multiple biological pathways. Our findings provide meaningful insights into issues related to immunotherapy response assessment, prognosis prediction, and development of potential prognostic biomarkers in patients with LUAD.

## Figures and Tables

**Table 1:** Clinical information of the TCGA-LUAD cohort.

Clinical Features	Number of Patients			*p*-Value
	Overall	Testing Set	Training Set	
All patients	476	136	340	
OS State				0.9169
Alive	294 (61.76)	85 (62.50)	209 (61.47)	
Dead	182 (38.24)	51 (37.50)	131 (38.53)	
Age	66.5 (59, 72)	66 (59, 73)	67 (59, 72)	0.7982
Gender				0.3447
FEMALE	256 (53.78)	68 (50.00)	188 (55.29)	
MALE	220 (46.22)	68 (50.00)	152 (44.71)	
Stage				0.5346
Stage I	253 (54.06)	69 (51.88)	184 (54.93)	
Stage II	114 (24.36)	37 (27.82)	77 (22.99)	
Stage III	75 (16.03)	22 (16.54)	53 (15.82)	
Stage IV	26 (5.56)	5 (3.76)	21 (6.27)	
No staging information	8 (1.68)	3 (2.21)	5 (1.47)	

## Data Availability

All data and R scripts used in this study can be requested from the corresponding author with due cause. TCGA (https://portal.gdc.cancer.gov, accessed on 1 May 2023), ImmPort (https://www.immport.org/home, accessed 1 May 2023), TCIA (https://tcia.at, accessed 1 May 2023), and GEO (https://www.ncbi.nlm.nih.gov/geo/, accessed 2 May 2023) furnished the public data used in this study. R software (version 4.1.3, available at https://www.r-project.org, accessed 3 March 2023) was utilized in this work to analyze the data in terms of bioinformatics and to visualize the outcomes.

## Supplementary Materials

The following supporting information can be downloaded at: https://www.mdpi.com/article/10.3390/life13071583/s1. Figure S1. Consensus clustering results and differences of gene expression levels. (A–G) Consensus clustering mapping for k from 3 to 9. (H) Delta plot of the relative change in k and k-1 areas under the CDF curve. Visualization of (I) PCoA and (J) tSNE for 17 cuproptosis-associated IRGs. (K) Volcano map of genes differentially expressed in both subtypes. Figure S2. Atlas of immunoinfiltrating cells. (A) Heatmap revealing the abundance of immunoinfiltrating cells of both subtypes calculated by ssGSEA. (B) Correlation of 17 cuproptosis-related IRGs with 28 immune cells. (C,D) xCell calculating abundance of 64 tumor-infiltrating lymphocytes (TILs), immune and stromal scores. Abundance of TILs calculated by the (E) CIBERSORT, (F) MCP-counter, (G) TIMER2.0. Error bar indicates confidence interval and point indicates median value. “ns”: not significant; “*”: *p* < 0.05; “**”: *p* < 0.01; “***”: *p* < 0.001; “****”: *p* < 0.0001. Figure S3. Assessment of the robustness and stability of the risk model. (A) Kaplan–Meier curves of both subgroups in the whole cohort. (B) Risk factor linkage plots in whole cohort demonstrate the status of patient survival and expression levels of signature genes in response to risk scores. Correlation between expression of (C) NRAS, (D) TRAV38-2DV8, and (E) SORT1, and risk scores. (F,G) Difference in risk scores by clinical stage and gender. (H–K) Kaplan–Meier curves for the two subgroups according to clinical stage and gender. Figure S4. Bubble map of marker genes for cell types. Red indicates upregulated expression and blue indicates downregulated expression. The size of the circles indicates the proportion of cells expressing the gene in each cell type. Figure S5. Correlation of risk scores and biological processes. (A) Scatter plot of correlation between risk scores and 14 pathways. (B) Heatmap of the correlation between risk scores and 14 pathways. Table S1. Results of coexpression analysis of cuproptosis-related genes and IRGs. Table S2. List of gene sets enriched in GSVA. Table S3. List of gene sets including 14 biological processes. Table S4. Univariate and multivariate Cox regression analyses for risk stratification.

## References

[1] Sung H., Ferlay J., Siegel R.L., Laversanne M., Soerjomataram I., Jemal A., Bray F. (2021). Global Cancer Statistics 2020: GLOBOCAN Estimates of Incidence and Mortality Worldwide for 36 Cancers in 185 Countries. CA Cancer J. Clin..

[2] Molina J.R., Yang P., Cassivi S.D., Schild S.E., Adjei A.A. (2008). Non-small cell lung cancer: Epidemiology, risk factors, treatment, and survivorship. Mayo Clin. Proc..

[3] Yi M., Li A., Zhou L., Chu Q., Luo S., Wu K. (2021). Immune signature-based risk stratification and prediction of immune checkpoint inhibitor’s efficacy for lung adenocarcinoma. Cancer Immunol. Immunother..

[4] Hanna N.H., Schneider B.J., Temin S., Baker S., Brahmer J., Ellis P.M., Gaspar L.E., Haddad R.Y., Hesketh P.J., Jain D. (2020). Therapy for Stage IV Non-Small-Cell Lung Cancer Without Driver Alterations: ASCO and OH (CCO) Joint Guideline Update. J. Clin. Oncol..

[5] Yi M., Qin S., Zhao W., Yu S., Chu Q., Wu K. (2018). The role of neoantigen in immune checkpoint blockade therapy. Exp. Hematol. Oncol..

[6] Ettinger D.S., Wood D.E., Aisner D.L., Akerley W., Bauman J.R., Bharat A., Bruno D.S., Chang J.Y., Chirieac L.R., D’Amico T.A. (2022). Non-Small Cell Lung Cancer, Version 3.2022, NCCN Clinical Practice Guidelines in Oncology. J. Natl. Compr. Cancer Netw..

[7] Passaro A., Brahmer J., Antonia S., Mok T., Peters S. (2022). Managing Resistance to Immune Checkpoint Inhibitors in Lung Cancer: Treatment and Novel Strategies. J. Clin. Oncol..

[8] Yang S.R., Schultheis A.M., Yu H., Mandelker D., Ladanyi M., Büttner R. (2022). Precision medicine in non-small cell lung cancer: Current applications and future directions. Semin. Cancer Biol..

[9] Gibney G.T., Weiner L.M., Atkins M.B. (2016). Predictive biomarkers for checkpoint inhibitor-based immunotherapy. Lancet Oncol..

[10] Saporito-Magriñá C., Musacco-Sebio R., Acosta J.M., Bajicoff S., Paredes-Fleitas P., Reynoso S., Boveris A., Repetto M.G. (2017). Copper(II) and iron(III) ions inhibit respiration and increase free radical-mediated phospholipid peroxidation in rat liver mitochondria: Effect of antioxidants. J. Inorg. Biochem..

[11] Sha S., Si L., Wu X., Chen Y., Xiong H., Xu Y., Liu W., Mei H., Wang T., Li M. (2022). Prognostic analysis of cuproptosis-related gene in triple-negative breast cancer. Front. Immunol..

[12] Kaplan J.H., Maryon E.B. (2016). How Mammalian Cells Acquire Copper: An Essential but Potentially Toxic Metal. Biophys. J..

[13] Hu G.F. (1998). Copper stimulates proliferation of human endothelial cells under culture. J. Cell. Biochem..

[14] McAuslan B.R., Reilly W. (1980). Endothelial cell phagokinesis in response to specific metal ions. Exp. Cell Res..

[15] Pan Q., Kleer C.G., van Golen K.L., Irani J., Bottema K.M., Bias C., De Carvalho M., Mesri E.A., Robins D.M., Dick R.D. (2002). Copper deficiency induced by tetrathiomolybdate suppresses tumor growth and angiogenesis. Cancer Res..

[16] Voli F., Valli E., Lerra L., Kimpton K., Saletta F., Giorgi F.M., Mercatelli D., Rouaen J.R.C., Shen S., Murray J.E. (2020). Intratumoral Copper Modulates PD-L1 Expression and Influences Tumor Immune Evasion. Cancer Res..

[17] Jiang Y., Huo Z., Qi X., Zuo T., Wu Z. (2022). Copper-induced tumor cell death mechanisms and antitumor theragnostic applications of copper complexes. Nanomedicine.

[18] Tsvetkov P., Coy S., Petrova B., Dreishpoon M., Verma A., Abdusamad M., Rossen J., Joesch-Cohen L., Humeidi R., Spangler R.D. (2022). Copper induces cell death by targeting lipoylated TCA cycle proteins. Science.

[19] Wu J., Zhao Y., Zhang J., Wu Q., Wang W. (2019). Development and validation of an immune-related gene pairs signature in colorectal cancer. Oncoimmunology.

[20] Shen S., Wang G., Zhang R., Zhao Y., Yu H., Wei Y., Chen F. (2019). Development and validation of an immune gene-set based Prognostic signature in ovarian cancer. EBioMedicine.

[21] Dai Y., Qiang W., Lin K., Gui Y., Lan X., Wang D. (2021). An immune-related gene signature for predicting survival and immunotherapy efficacy in hepatocellular carcinoma. Cancer Immunol. Immunother..

[22] Kalinke L., Janes S.M. (2022). Two phenotypes that predict prognosis in lung adenocarcinoma. Eur. Respir. J..

[23] Bhattacharya S., Dunn P., Thomas C.G., Smith B., Schaefer H., Chen J., Hu Z., Zalocusky K.A., Shankar R.D., Shen-Orr S.S. (2018). ImmPort, toward repurposing of open access immunological assay data for translational and clinical research. Sci. Data.

[24] Charoentong P., Finotello F., Angelova M., Mayer C., Efremova M., Rieder D., Hackl H., Trajanoski Z. (2017). Pan-cancer Immunogenomic Analyses Reveal Genotype-Immunophenotype Relationships and Predictors of Response to Checkpoint Blockade. Cell Rep..

[25] Wilkerson M.D., Hayes D.N. (2010). ConsensusClusterPlus: A class discovery tool with confidence assessments and item tracking. Bioinformatics.

[26] Hänzelmann S., Castelo R., Guinney J. (2013). GSVA: Gene set variation analysis for microarray and RNA-seq data. BMC Bioinform..

[27] Becht E., Giraldo N.A., Lacroix L., Buttard B., Elarouci N., Petitprez F., Selves J., Laurent-Puig P., Sautès-Fridman C., Fridman W.H. (2016). Estimating the population abundance of tissue-infiltrating immune and stromal cell populations using gene expression. Genome Biol..

[28] Aran D., Hu Z., Butte A.J. (2017). xCell: Digitally portraying the tissue cellular heterogeneity landscape. Genome Biol..

[29] Newman A.M., Liu C.L., Green M.R., Gentles A.J., Feng W., Xu Y., Hoang C.D., Diehn M., Alizadeh A.A. (2015). Robust enumeration of cell subsets from tissue expression profiles. Nat. Methods.

[30] Yoshihara K., Shahmoradgoli M., Martínez E., Vegesna R., Kim H., Torres-Garcia W., Treviño V., Shen H., Laird P.W., Levine D.A. (2013). Inferring tumour purity and stromal and immune cell admixture from expression data. Nat. Commun..

[31] Friedman J., Hastie T., Tibshirani R. (2010). Regularization Paths for Generalized Linear Models via Coordinate Descent. J. Stat. Softw..

[32] Zhang Z., Cortese G., Combescure C., Marshall R., Lee M., Lim H.J., Haller B. (2018). Overview of model validation for survival regression model with competing risks using melanoma study data. Ann. Transl. Med..

[33] Blanche P., Dartigues J.F., Jacqmin-Gadda H. (2013). Estimating and comparing time-dependent areas under receiver operating characteristic curves for censored event times with competing risks. Stat. Med..

[34] Macosko E.Z., Basu A., Satija R., Nemesh J., Shekhar K., Goldman M., Tirosh I., Bialas A.R., Kamitaki N., Martersteck E.M. (2015). Highly Parallel Genome-wide Expression Profiling of Individual Cells Using Nanoliter Droplets. Cell.

[35] Huang Z.Y., Shao M.M., Zhang J.C., Yi F.S., Du J., Zhou Q., Wu F.Y., Li S., Li W., Huang X.Z. (2021). Single-cell analysis of diverse immune phenotypes in malignant pleural effusion. Nat. Commun..

[36] Wu F., Fan J., He Y., Xiong A., Yu J., Li Y., Zhang Y., Zhao W., Zhou F., Li W. (2021). Single-cell profiling of tumor heterogeneity and the microenvironment in advanced non-small cell lung cancer. Nat. Commun..

[37] Salcher S., Sturm G., Horvath L., Untergasser G., Kuempers C., Fotakis G., Panizzolo E., Martowicz A., Trebo M., Pall G. (2022). High-resolution single-cell atlas reveals diversity and plasticity of tissue-resident neutrophils in non-small cell lung cancer. Cancer Cell.

[38] He D., Wang D., Lu P., Yang N., Xue Z., Zhu X., Zhang P., Fan G. (2021). Single-cell RNA sequencing reveals heterogeneous tumor and immune cell populations in early-stage lung adenocarcinomas harboring EGFR mutations. Oncogene.

[39] Aran D., Looney A.P., Liu L., Wu E., Fong V., Hsu A., Chak S., Naikawadi R.P., Wolters P.J., Abate A.R. (2019). Reference-based analysis of lung single-cell sequencing reveals a transitional profibrotic macrophage. Nat. Immunol..

[40] Subramanian A., Tamayo P., Mootha V.K., Mukherjee S., Ebert B.L., Gillette M.A., Paulovich A., Pomeroy S.L., Golub T.R., Lander E.S. (2005). Gene set enrichment analysis: A knowledge-based approach for interpreting genome-wide expression profiles. Proc. Natl. Acad. Sci. USA.

[41] Mayakonda A., Lin D.C., Assenov Y., Plass C., Koeffler H.P. (2018). Maftools: Efficient and comprehensive analysis of somatic variants in cancer. Genome Res..

[42] Inamura K. (2018). Clinicopathological Characteristics and Mutations Driving Development of Early Lung Adenocarcinoma: Tumor Initiation and Progression. Int. J. Mol. Sci..

[43] (2014). Comprehensive molecular profiling of lung adenocarcinoma. Nature.

[44] Campbell J.D., Alexandrov A., Kim J., Wala J., Berger A.H., Pedamallu C.S., Shukla S.A., Guo G., Brooks A.N., Murray B.A. (2016). Distinct patterns of somatic genome alterations in lung adenocarcinomas and squamous cell carcinomas. Nat. Genet..

[45] She Y., Jin Z., Wu J., Deng J., Zhang L., Su H., Jiang G., Liu H., Xie D., Cao N. (2020). Development and Validation of a Deep Learning Model for Non-Small Cell Lung Cancer Survival. JAMA Netw. Open.

[46] Hinshaw D.C., Shevde L.A. (2019). The Tumor Microenvironment Innately Modulates Cancer Progression. Cancer Res..

[47] Wang L., Zhu B., Zhang M., Wang X. (2017). Roles of immune microenvironment heterogeneity in therapy-associated biomarkers in lung cancer. Semin. Cell Dev. Biol..

[48] Zhang C., Zeng Y., Guo X., Shen H., Zhang J., Wang K., Ji M., Huang S. (2022). Pan-cancer analyses confirmed the cuproptosis-related gene FDX1 as an immunotherapy predictor and prognostic biomarker. Front. Genet..

[49] Cai Y., He Q., Liu W., Liang Q., Peng B., Li J., Zhang W., Kang F., Hong Q., Yan Y. (2022). Comprehensive analysis of the potential cuproptosis-related biomarker LIAS that regulates prognosis and immunotherapy of pan-cancers. Front. Oncol..

[50] Lv H., Liu X., Zeng X., Liu Y., Zhang C., Zhang Q., Xu J. (2022). Comprehensive Analysis of Cuproptosis-Related Genes in Immune Infiltration and Prognosis in Melanoma. Front. Pharmacol..

[51] Liu Z., Yu M., Fei B., Fang X., Ma T., Wang D. (2018). miR-21-5p targets PDHA1 to regulate glycolysis and cancer progression in gastric cancer. Oncol. Rep..

[52] Sun S., Guo W., Wang Z., Wang X., Zhang G., Zhang H., Li R., Gao Y., Qiu B., Tan F. (2020). Development and validation of an immune-related prognostic signature in lung adenocarcinoma. Cancer Med..

[53] Wu C., Hu Q., Ma D. (2021). Development of an immune-related gene pairs signature for predicting clinical outcome in lung adenocarcinoma. Sci. Rep..

[54] Shi X., Li R., Dong X., Chen A.M., Liu X., Lu D., Feng S., Wang H., Cai K. (2020). IRGS: An immune-related gene classifier for lung adenocarcinoma prognosis. J. Transl. Med..

[55] Ma K.Y., Schonnesen A.A., Brock A., Van Den Berg C., Eckhardt S.G., Liu Z., Jiang N. (2019). Single-cell RNA sequencing of lung adenocarcinoma reveals heterogeneity of immune response-related genes. JCI Insight.

[56] Wu K., Lin K., Li X., Yuan X., Xu P., Ni P., Xu D. (2020). Redefining Tumor-Associated Macrophage Subpopulations and Functions in the Tumor Microenvironment. Front. Immunol..

[57] Smyth M.J., Crowe N.Y., Godfrey D.I. (2001). NK cells and NKT cells collaborate in host protection from methylcholanthrene-induced fibrosarcoma. Int. Immunol..

[58] Girardi M., Oppenheim D.E., Steele C.R., Lewis J.M., Glusac E., Filler R., Hobby P., Sutton B., Tigelaar R.E., Hayday A.C. (2018). Pillars Article: Regulation of Cutaneous Malignancy by γδ T Cells. Science. 2001. 294: 605–609. J. Immunol..

[59] Zhao W., Zhu B., Hutchinson A., Pesatori A.C., Consonni D., Caporaso N.E., Zhang T., Wang D., Shi J., Landi M.T. (2022). Clinical Implications of Inter- and Intratumor Heterogeneity of Immune Cell Markers in Lung Cancer. J. Natl. Cancer Inst..

[60] Pilch Z., Tonecka K., Braniewska A., Sas Z., Skorzynski M., Boon L., Golab J., Meyaard L., Rygiel T.P. (2018). Antitumor Activity of TLR7 Is Potentiated by CD200R Antibody Leading to Changes in the Tumor Microenvironment. Cancer Immunol. Res..

[61] Dajon M., Iribarren K., Cremer I. (2015). Dual roles of TLR7 in the lung cancer microenvironment. Oncoimmunology.

[62] Mei J., Jiang G., Chen Y., Xu Y., Wan Y., Chen R., Liu F., Mao W., Zheng M., Xu J. (2022). HLA class II molecule HLA-DRA identifies immuno-hot tumors and predicts the therapeutic response to anti-PD-1 immunotherapy in NSCLC. BMC Cancer.

[63] Fling S.P., Arp B., Pious D. (1994). HLA-DMA and -DMB genes are both required for MHC class II/peptide complex formation in antigen-presenting cells. Nature.

[64] Yan Y., Gao Z., Han H., Zhao Y., Zhang Y., Ma X., Chen H. (2022). NRAS expression is associated with prognosis and tumor immune microenvironment in lung adenocarcinoma. J. Cancer Res. Clin. Oncol..

[65] Giannou A.D., Marazioti A., Kanellakis N.I., Giopanou I., Lilis I., Zazara D.E., Ntaliarda G., Kati D., Armenis V., Giotopoulou G.A. (2017). NRAS destines tumor cells to the lungs. EMBO Mol. Med..

[66] Gao Y., Li Y., Song Z., Jin Z., Li X., Yuan C. (2022). Sortilin 1 Promotes Hepatocellular Carcinoma Cell Proliferation and Migration by Regulating Immune Cell Infiltration. J. Oncol..

[67] Liang M., Yao W., Shi B., Zhu X., Cai R., Yu Z., Guo W., Wang H., Dong Z., Lin M. (2021). Circular RNA hsa_circ_0110389 promotes gastric cancer progression through upregulating SORT1 via sponging miR-127-5p and miR-136-5p. Cell Death Dis..

[68] Johnson I.R., Parkinson-Lawrence E.J., Keegan H., Spillane C.D., Barry-O’Crowley J., Watson W.R., Selemidis S., Butler L.M., O’Leary J.J., Brooks D.A. (2015). Endosomal gene expression: A new indicator for prostate cancer patient prognosis?. Oncotarget.

[69] Blondy S., Talbot H., Saada S., Christou N., Battu S., Pannequin J., Jauberteau M.O., Lalloué F., Verdier M., Mathonnet M. (2021). Overexpression of sortilin is associated with 5-FU resistance and poor prognosis in colorectal cancer. J. Cell. Mol. Med..

[70] Iasonos A., Schrag D., Raj G.V., Panageas K.S. (2008). How to build and interpret a nomogram for cancer prognosis. J. Clin. Oncol..

