# Supplementary material for: Cuproptosis and Immune-Related Gene Signature Predicts Immunotherapy Response and Prognosis in Lung Adenocarcinoma
Source: Life (Basel). 2023 Jul 19;13(7):1583. doi: 10.3390/life13071583 (PMC10381686; doi:10.3390/life13071583)
Supplement: Supplementary file 1 [file life-13-01583-s001.zip › Supplementary Figure S1.pdf]

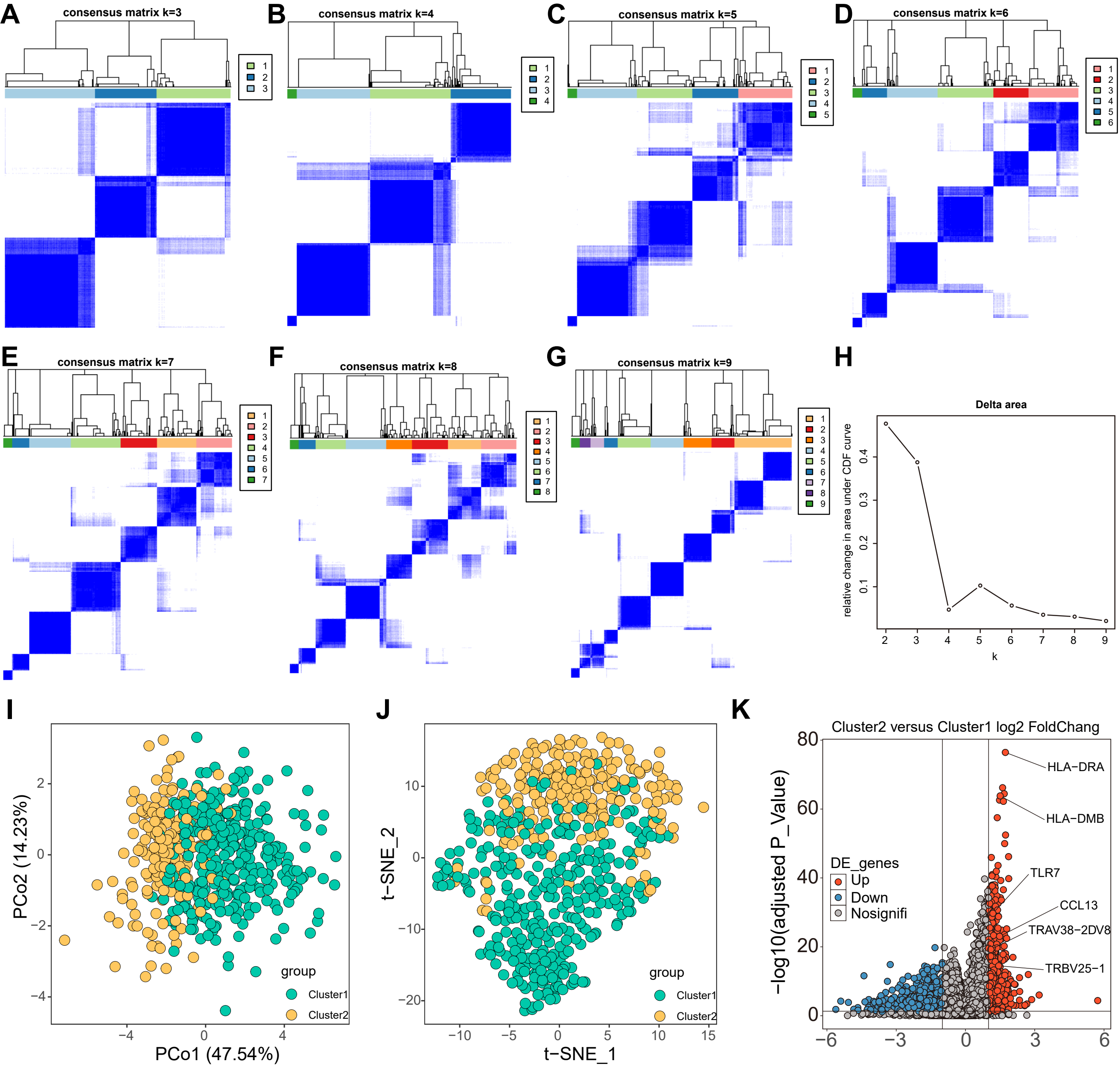

Figure S1. Consensus clustering results and differences of gene expression levels. (A–G) Consensus clustering mapping for k from 3 to 9. (H) Delta plot of the relative change in k and k-1 areas under the CDF curve. Visualization of (I) PCoA and (J) tSNE for 17 cuproptosis-associated IRGs. (K) Volcano map of genes differentially expressed in both subtypes.
