# Supplementary material for: Cuproptosis and Immune-Related Gene Signature Predicts Immunotherapy Response and Prognosis in Lung Adenocarcinoma
Source: Life (Basel). 2023 Jul 19;13(7):1583. doi: 10.3390/life13071583 (PMC10381686; doi:10.3390/life13071583)
Supplement: Supplementary file 1 [file life-13-01583-s001.zip › Supplementary Figure S2.pdf]

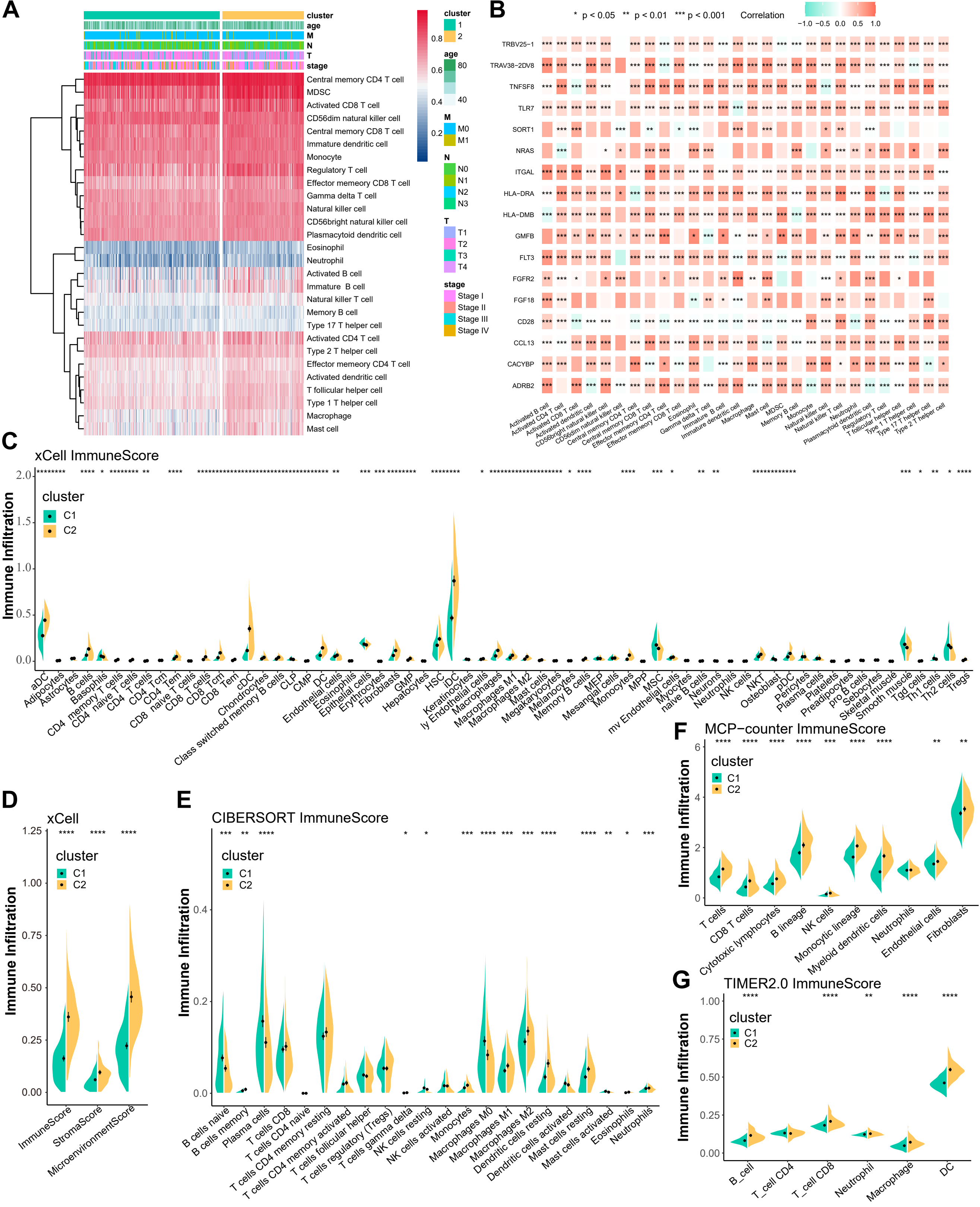

Figure S2. Atlas of immunoinfiltrating cells.
(A) Heatmap revealing the abundance of immunoinfiltrating cells of both subtypes calculated by ssGSEA. (B) Correlation of 17 cuproptosis-related IRGs with 28 immune cells. (C,D) xCell calcu-lating abundance of 64 tumor-infiltrating lymphocytes (TILs), immune and stromal scores. Abun-dance of TILs calculated by the (E) CIBERSORT, (F) MCP-counter, (G) TIMER2.0. Error bar indi-cates confidence interval and point indicates median value. “*”: $p < 0.05$; “**”: $p < 0.01$; “***”: $p < 0.001$; “****”: $p < 0.0001$.
