# Supplementary material for: Cuproptosis and Immune-Related Gene Signature Predicts Immunotherapy Response and Prognosis in Lung Adenocarcinoma
Source: Life (Basel). 2023 Jul 19;13(7):1583. doi: 10.3390/life13071583 (PMC10381686; doi:10.3390/life13071583)
Supplement: Supplementary file 1 [file life-13-01583-s001.zip › Supplementary Figure S3.pdf]

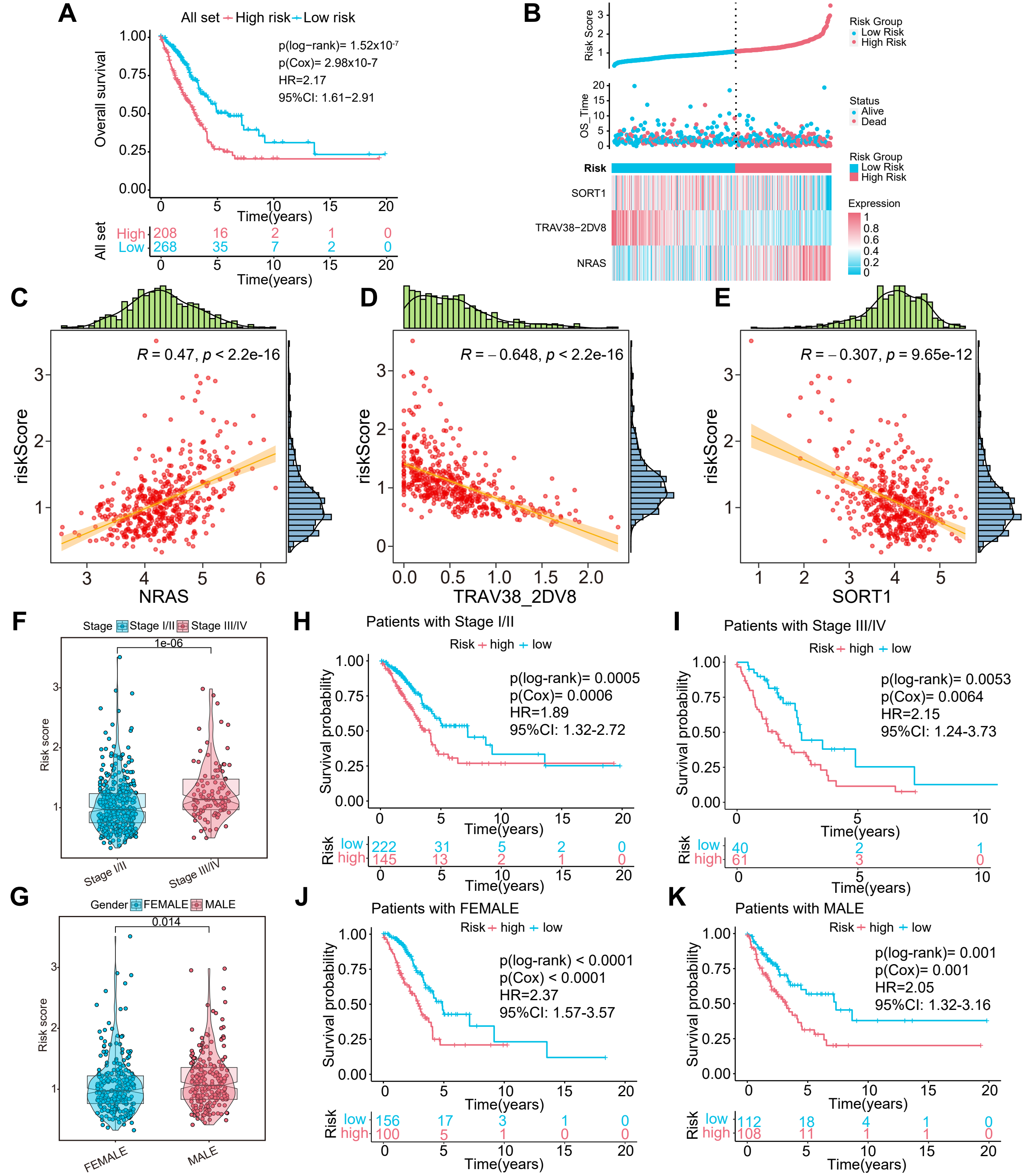


Figure S3. Assessment of the robustness and stability of the risk model.
(A) Kaplan–Meier curves of both subgroups in the whole cohort. (B) Risk factor linkage plots in whole cohort demonstrate the status of patient survival and expression levels of signature genes in response to risk scores. Correlation between expression of (C) NRAS, (D) TRAV38-2DV8, and (E) SORT1, and risk scores. (F,G) Difference in risk scores by clinical stage and gender. (H–K) Kaplan–Meier curves for the two subgroups according to clinical stage and gender.
