# Supplementary material for: Cuproptosis and Immune-Related Gene Signature Predicts Immunotherapy Response and Prognosis in Lung Adenocarcinoma
Source: Life (Basel). 2023 Jul 19;13(7):1583. doi: 10.3390/life13071583 (PMC10381686; doi:10.3390/life13071583)
Supplement: Supplementary file 1 [file life-13-01583-s001.zip › Supplementary Figure S4.pdf]

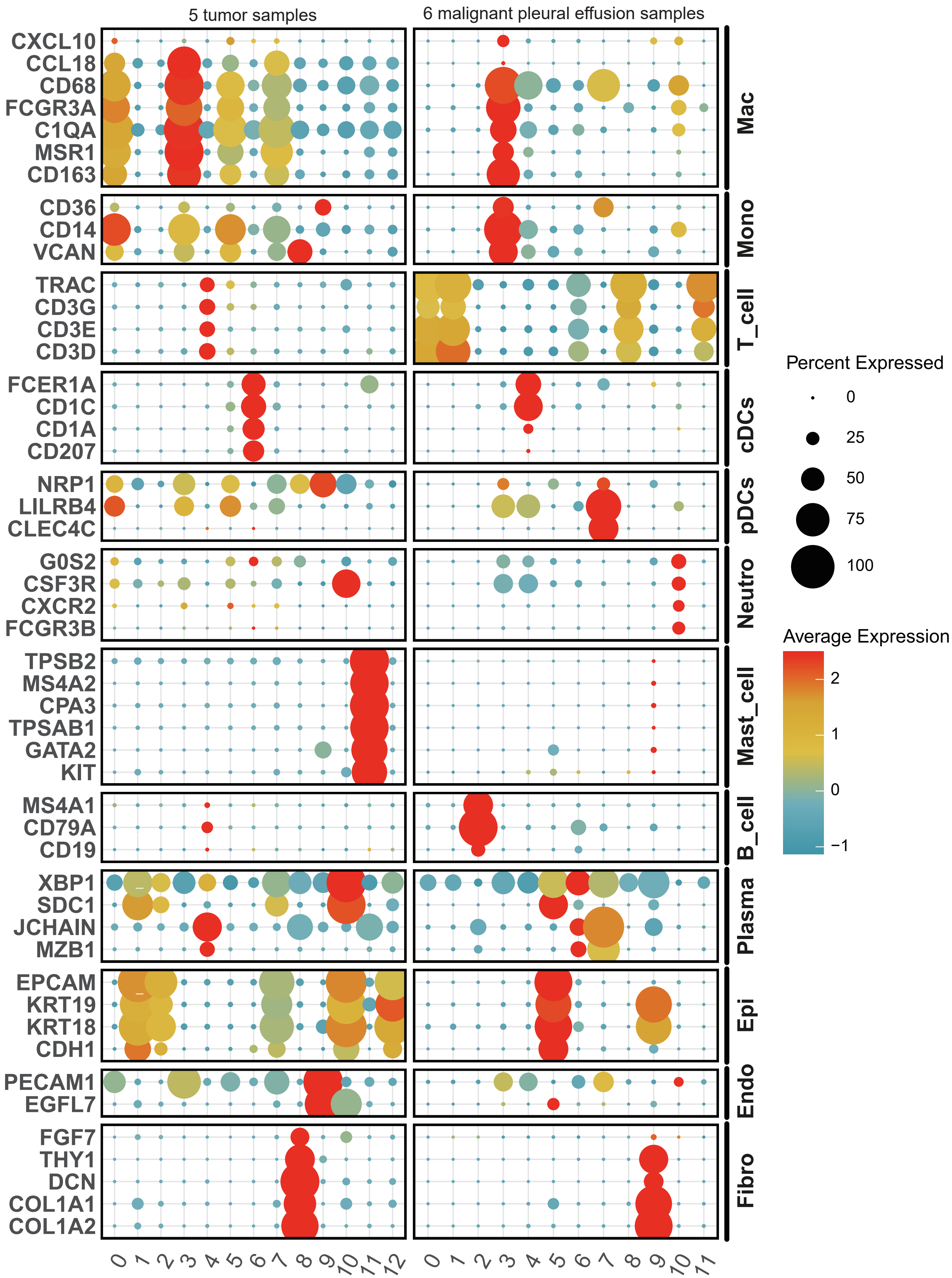

Figure S4. Bubble map of marker genes for cell types.
Red indicates upregulated expression and blue indicates downregulated expression. The size of the circles indicates the proportion of cells expressing the gene in each cell type.
