# Supplementary material for: Cuproptosis and Immune-Related Gene Signature Predicts Immunotherapy Response and Prognosis in Lung Adenocarcinoma
Source: Life (Basel). 2023 Jul 19;13(7):1583. doi: 10.3390/life13071583 (PMC10381686; doi:10.3390/life13071583)
Supplement: Supplementary file 1 [file life-13-01583-s001.zip › Supplementary Figure S5.pdf]

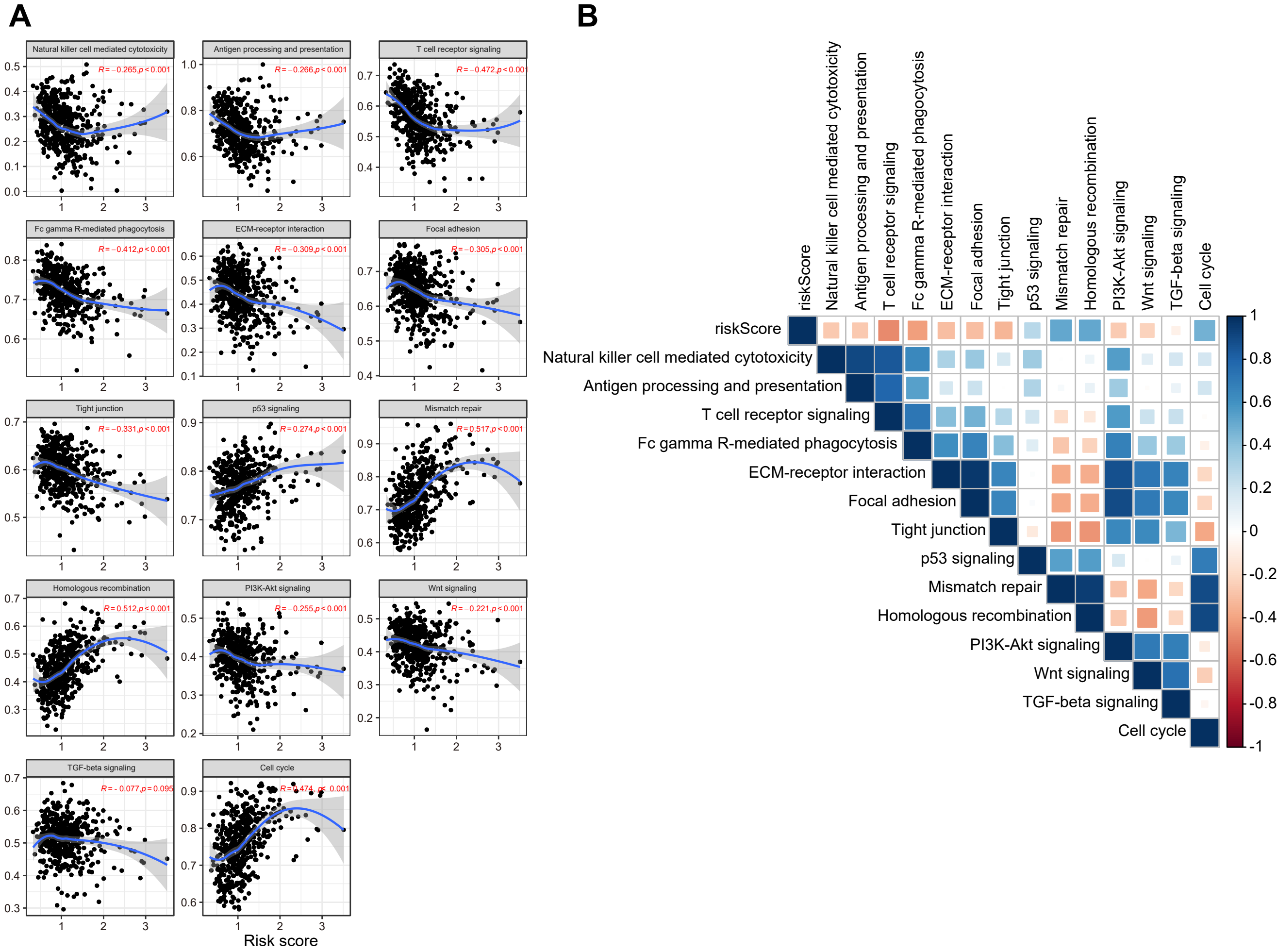

Figure S5. Correlation of risk scores and biological processes.
(A) Scatter plot of correlation be-tween risk scores and 14 pathways. (B) Heatmap of the correlation between risk scores and 14 pathways.
