# Supplementary material for: Cuproptosis and Immune-Related Gene Signature Predicts Immunotherapy Response and Prognosis in Lung Adenocarcinoma
Source: Life (Basel). 2023 Jul 19;13(7):1583. doi: 10.3390/life13071583 (PMC10381686; doi:10.3390/life13071583)
Supplement: Supplementary file 1 [file life-13-01583-s001.zip › Supplementary Table 1.pdf]

Table S1. Results of coexpression analysis of cuproptosis-related genes and IRGs.

Cuproptosis-related genes	Immune-related genes	cor	pvalue	Regulation
**NLRP3**	**ITGAL**	0.434584547	3.48E-25	postive
**NLRP3**	**TYROBP**	0.412525295	1.28E-22	postive
**NLRP3**	**MSR1**	0.427298943	2.57E-24	postive
**NFE2L2**	**FGFR2**	0.55357778	9.09E-43	postive
**FDX1**	**TGFBR3**	0.662955034	1.28E-66	postive
**MTF1**	**TXLNA**	0.533796855	2.42E-39	postive
**NLRP3**	**OSM**	0.353315499	1.29E-16	postive
**NLRP3**	**TNFSF13B**	0.433787281	4.34E-25	postive
**ATP7A**	**NFAT5**	0.400672107	2.56E-21	postive
**MTF1**	**NFAT5**	0.4294612	1.43E-24	postive
**NLRP3**	**EBI3**	0.437474385	1.55E-25	postive
**DLD**	**NAMPT**	0.379764186	3.79E-19	postive
**DLD**	**LMBR1**	0.386257761	8.34E-20	postive
**FDX1**	**TGFBR1**	0.457623871	4.52E-28	postive
**NLRP3**	**TNFSF8**	0.606156929	4.44E-53	postive
**MTF1**	**CBL**	0.518226759	8.39E-37	postive
**NLRP3**	**CD86**	0.655635868	1.04E-64	postive
**MTF1**	**SOS1**	0.529860511	1.09E-38	postive
**PDHB**	**CACYBP**	0.357385946	5.44E-17	postive
**MTF1**	**NENF**	-0.4034557	1.28E-21	negative
**NFE2L2**	**TNFSF18**	0.384870938	1.16E-19	postive
**NLRP3**	**FLT3**	0.402682243	1.55E-21	postive
**NLRP3**	**LRP1**	0.360734943	2.65E-17	postive
**NLRP3**	**IL1B**	0.555436388	4.21E-43	postive
**PDHB**	**TPT1**	0.379989505	3.60E-19	postive
**MTF1**	**TPT1**	-0.460039541	2.18E-28	negative
**DBT**	**SORT1**	0.375369322	1.04E-18	postive
**NLRP3**	**IL2RA**	0.65960403	9.71E-66	postive
**LIPT1**	**TANK**	0.414876419	6.94E-23	postive

**NLRP3**	**IL10**	0.390320714	3.18E-20	postive
**PDHB**	**MANF**	0.392541615	1.86E-20	postive
**FDX1**	**CRIM1**	0.575998733	6.16E-47	postive
**ATP7B**	**FGF18**	0.437313843	1.63E-25	postive
**NLRP3**	**FCER1G**	0.553923465	7.88E-43	postive
**NLRP3**	**CCR1**	0.452794511	1.90E-27	postive
**NLRP3**	**CTSB**	0.4082612	3.80E-22	postive
**NLRP3**	**CYBB**	0.641472582	3.67E-61	postive
**NLRP3**	**ADRB2**	0.374269249	1.33E-18	postive
**NLRP3**	**RNASE2**	0.388051979	5.45E-20	postive
**NLRP3**	**CD14**	0.604455641	1.03E-52	postive
**NLRP3**	**S1PR1**	0.387165432	6.73E-20	postive
**NLRP3**	**FPR2**	0.584341071	1.43E-48	postive
**NLRP3**	**FPR1**	0.673129864	2.28E-69	postive
**NLRP3**	**C3AR1**	0.712522464	3.97E-81	postive
**NLRP3**	**TLR1**	0.422405954	9.57E-24	postive
**FDX1**	**LEP**	0.752740396	2.21E-95	postive
**LIPT1**	**CRLF3**	0.365653185	9.09E-18	postive
**NLRP3**	**IL17RA**	0.370428221	3.16E-18	postive
**MTF1**	**IL17RA**	0.38546818	1.00E-19	postive
**NLRP3**	**CD28**	0.531684754	5.44E-39	postive
**DLAT**	**PTPN11**	0.397422283	5.69E-21	postive
**MTF1**	**PTPN11**	0.362127382	1.96E-17	postive
**NLRP3**	**CCL13**	0.423567746	7.02E-24	postive
**NLRP3**	**CSF1R**	0.707821793	1.27E-79	postive
**PDHB**	**PLXNB2**	-0.355083	8.87E-17	negative
**NLRP3**	**TLR7**	0.536252304	9.33E-40	postive
**DLST**	**GMFB**	0.393604763	1.44E-20	postive
**MTF1**	**IGF2R**	0.456443176	6.43E-28	postive
**NLRP3**	**CSF2RA**	0.437154587	1.70E-25	postive

**NLRP3**	**FCGR3A**	0.540674229	1.65E-40	postive
**NLRP3**	**HLA-DRA**	0.440882952	5.95E-26	postive
**NLRP3**	**TRAV38-2DV8**	0.435937892	2.39E-25	postive
**DLAT**	**NRAS**	0.467967506	1.93E-29	postive
**DLD**	**CHUK**	0.372309969	2.07E-18	postive
**DLAT**	**CHUK**	0.473470705	3.45E-30	postive
**NLRP3**	**HLA-DMB**	0.431447852	8.28E-25	postive
**MTF1**	**CNTF**	0.363877189	1.34E-17	postive
**NLRP3**	**CCL3**	0.364111086	1.27E-17	postive
**NLRP3**	**TRBV25-1**	0.472285858	5.01E-30	postive
