# Supplementary material for: Cuproptosis and Immune-Related Gene Signature Predicts Immunotherapy Response and Prognosis in Lung Adenocarcinoma
Source: Life (Basel). 2023 Jul 19;13(7):1583. doi: 10.3390/life13071583 (PMC10381686; doi:10.3390/life13071583)
Supplement: Supplementary file 1 [file life-13-01583-s001.zip › Supplementary Table 2.pdf]

Table S2. List of gene sets enriched in GSVA.

Source	Gene Signature	associated genes
KEGG hsa04115	p53 signaling pathway	CCNB2, CCNE1, CCNE2, CDK1, CDK2, CDKN2A, GTSE1, PPM1D, RFWD2, RRM2
KEGG ocu04060	Cytokine-cytokine receptor interaction	ACVR1, CCL24, FLT4, IFNGR1, IL4R, IL6ST, KIT, LIF, PDGFA, PDGFRB, TGFB1, TGFBR2, TNFRSF10B, TNFRSF14, TNFRSF1A
PMID: 26855148	AR_signaling	MPHOSPH9, ADAM7, FOLH1, CD200, FKBP5, GLRA2, NDRG1, CAMKK2, MAN1A1, MED28, ELL2, ACSL3, PMEPA1, GNMT, ABCC4, HERC3, PIP4K2B, KLK3, EAF2, CENPN, MAPRE2, NKX3-1, KLK2, AR, TNK1, MAF, C1ORF116, TMPRSS2, TBC1D9B, ZBTB10
MSigDB	Wong_SCs	RPL13, RPS8, HELLS, RCN2, DLGAP5, EMC8, POLE2, EIF3K, FGFR1, LSM4, RPS3, MRPL15, VRK1, HNRNPL, EIF4A1, KIF22, NUP107, PIPOX, HAUS1, THOC3, RFC3, NTHL1, CCNF, PPP4C, AURKB, PABPC1, HSPA14, MYBL2, FBL, CDCA7, HSPE1, MRPS30, MSH2, BUB1, CKAP2, GLDC, AK4, HMGN5, TERF1, EIF6, PSMD14, NDUFB8, ALDH7A1, RUVBL2, NAP1L1, RPS16, EBNA1BP2, MRPL37, DAP3, NDC80, MRPS36, G3BP1, NDUFB10, NHP2, TCF19, WEE1, RRM2, NDUFAB1, GTSE1, TIMM8A, CDCA8, RNPS1, PPM1G, UTP18, ETFA, KPNA6, HAT1, NEK2, RRM1, XRCC5, MRPL4, ZNF22, SLC16A1, HNRNPA1, NDUFB7, PHF5A, PUS1, MAPK13, FDPS, NUDCD2, BANF1, GNL3, RCC1, MCM5, CKS1B, CCNA2, RCC2, NONO, CDC34, LMNB1, DNMT1, MRPS28, FARSA, POLR2F, CLPP, DLAT, TEAD2, PSMA7, MRPL13, NCL, ELOVL6, HADH, SNRPA1, DPP3, PRIM2, PHC1, SOX2, CRABP2, PRMT3, RPS27, MRPL16, TP53, PSME3, NDUFS2, YAP1, NOP2, ATP5PO, GLO1, ECHS1, EIF4B, DBF4, HMGB2, ANP32E, PRMT1, MID1IP1, MCM2, CDC6, MCM3, U2AF1, POLR3K, RPS19, SRSF10, MAIP1, SS18, IARS1, PROM1, SMC2, UQCRH, ATP5PF, PSMB6, UQCR11, SNX5, AMOTL2, LBHD1, MYC, ENO1, SDHC, HDAC1, LSM10, RPL10A, DHX9, CISD1, UBE2V2, KIF20A, CCT5, PLK4, AURKA, ADH5, SDHD, CHAF1A, TGIF1, TTK, KIF11, RPS12, RPS5, SLC25A5, EIF4EBP1, EIF3I, GNA14, ABCB7, TCOF1, RUVBL1, VBP1, EEF2, DTYMK, PRDX1, SUMO1, CDK1, RAD18, SLC2A1, BIRC5, PCNA, TCF7L1, MRTO4, EIF2S2, LSM5, CCNC, SEPHS2, TRIP6, KIF4A, CTSC, MRPL39, TIMM8B, CDC20, MTF2, BLM, NIP7, BAX, PSMA5, TMEFF1, ALDOC, NOP10, TIMM44, ZIC3, MRPS18B, NIPSNAP1, PA2G4, KRAS, LYPLA1, BCAT1, SNRNP40, DEK, OTX2, CYCS, NDUFA11, CDC7, NCAPD2, GARS1, NME2, ERCC6L, ERP29, EIF3A, CBX3, WBP11, RACGAP1, PLK1, NLN, CDKN1C, NCAPH, DDX18, GSPT2, HNRNPK, SET, MAD2L1, NCBP2, PARP1, PDHA1, SNRPA, EIF3L, CDK4, FH, EXO1, YY1, XPO1, CCND1, IPO9, UGDH, KPNA2, FAM136A, NIFK, CDCA5, PRPS1, CSE1L, E2F3, CSRP2, TRIP13, MTHFD2, NUSAP1, NDUFA9, MRPL12, GNPDA1, PSMB5, PRIM1, LSM2, JPT1, NASP, SSB, CHEK2, BTF3, GART, SMC4, RPL22, CCND2, PHB, RPSA, RPS23, MCM7, GEMIN2, NT5DC2, MRPS2, RPP40, SARS1, PDIA4, UBE2G1, EIF2S3, WDHD1, CTNNA1, DARS2, TGIF2, COX5B, CDCA3, MRPS17, ORC1, GMNN, TIMM13, GJA1, STIP1, HSPA9, POP7, KIF23, GEMIN6, RPA3, RPA2, EXOSC7, CHEK1, RPL27A, CDKN3, BUB1B, HNRNPAB, SNRPD1, NME4, RAB34, BRIX1, EEF1E1, COQ3, APEX1, RAD23B, WDR77, ACAD8, MRPL11, TOP2A, SERPINH1, STOML2, CCNB2, MCM4, CKS2, BUB3, PDCD2, SPAG5, PDPN, POLD1, DTL, SINHCAF, ADSL, SQLE

MSigDB	RAMALHO_Stemness_UP	ZC3H14, RRN3, MPDU1, TXNDC9, ZNF644, MTMR10, TGS1, ZMAT3, UPP1, RCL1, STXBP3, COPS4, RAD23B, YWHAB, SMAD1, GLO1, BLZF1, PPP2R1B, ZNF101, BPNT2, LRRC58, YWHAH, USP10, SRSF6, URI1, CHD1, FASTKD5, TJP1, GARS1, MAP4K3, ROCK2, GAS2, SLC38A2, BACH1, SH3D19, DTYMK, SLC7A6, NIFK, YAP1, SUCLG2, FCF1, ZFX, LIG3, RIDA, ERCC5, LAS1L, GAB1, FBXO8, PACRGL, MDFIC, MRPS31, SAPCD1, PDCD2, FKBP11, LIMA1, ARCN1, UBE2T, MRPL45, RNFT1, ALDH7A1, LAPTM4A, UMPS, DICER1, CENPC, CDK2AP1, PSMD12, GRWD1, NDUFAF4, PPIC, MRPL17, MPHOSPH10, CRTAP, STAM, TEAD2, ZNF213, PCF11, XPO1, RYK, EIF3J, NOP58, GCAT, CPXM1, XRCC5, IARS2, COPRS, SRSF3, UTP4, RABGGTB, JAGN1, MDFI, PRPF6, MRPL34, PAFAH2, ZMYM4, TBC1D15, ZZZ3, LAPTM4B, MRPS2, CCND1, TCEAL9, GHR, ESF1, TGIF2-RAB5IF, DNAJB6, PSMD11, SEC23IP, MTERF3, TBRG4, ELOVL6, KCNAB3, EPRS1, SLC4A7, NUP35, ANKRD17, ACADM, RAB18, UTP20, TARS2, RARS2, TXNRD1, MARCHF7, USP9X, WDR43, PPP1R2, PTPN2, ELP2, RPP14, SEC23A, CTBP2, RPL22, SNX12, BYSL, RNF145, PLS3, ITGB1, C12orf45, ACAT2, RSRC2, PHTF2, FBXO38, RPUSD4, PRPSAP1, COPS7A, ARIH1, SMAD2, PPA1, RASA1, ZCCHC10, GCLM, CDKN1A, FHL1, EIF4G2, TOM1L1, TBRG1, F2R, PIGX, SMARCAD1, XPOT, INTS5, PLA2G6, TRIP6, LSG1, ZNF281, PKD2, CTTN, GSTA1, SNRPC, GFER, WDR55, DCTPP1, ZNF639, KANK3, CWC22, RNF138, MRPS10, IARS1, RNF4, RSL1D1, EIF4EBP1, TMEM183A, GNL2, FKBP9, SOCS2, C5orf51, RCN1, GNB1, ITGA6, GCSH, PEX7, MED23, ADAM9, LSM2, KIF2A, KRAS, ABCB1, YES1, DPH5
PMID: 31061129	2019_PNAS_stemness	DNMT3B, PFAS, XRCC5, HAUS6, TET1, IGF2BP1, PLAA, TEX10, MSH6, DLGAP5, SKIV2L2, SOHLH2, RRAS2, PAICS, CPSF3, LIN28B, IPO5, BMPR1A, ZNF788, ASCC3, FANCB, HMGA2, TRIM24, ORC1, HDAC2, HESX1, INHBE, MIS18A, DCUN1D5, MRPL3, CENPH, MYCN, HAUS1, GDF3, TBCE, RIOK2, BCKDHB, RAD1, NREP, ADH5, PLRG1, ROR1, RAB3B, DIAPH3, GNL2, FGF2, NMNAT2, KIF20A, CENPI, DDX1, XXYLT1, GPR176, BBS9, C14orf166, BOD1, CDC123, SNRPD3, FAM118B, DPH3, EIF2B3, RPF2, APLP1, DACT1, PDHB, C14orf119, DTD1, SAMM50, CCL26, MED20, UTP6, RARS2, ARMCX2, RARS, MTHFD2, DHX15, HTR7, MTHFD1L, ARMC9, XPOT, IARS, HDX, ACTRT3, ERCC2, TBC1D16, GARS, KIF7, UBE2K, SLC25A3, ICMT, UGGT2, ATP11C, SLC24A1, EIF2AK4, GPX8, ALX1, OSTC, TRPC4, HAS2, FZD2, TRNT1, MMADHC, SNX8, CDH6, HAT1, SEC11A, DIMT1, TM2D2, FST, GBE1
PMID: 22361632	Cell_cycle_progression	FOXM1, CDC20, CDKN3, CDC2, KIF11, KIAA0101, NUSAP1, CENPF, ASPM, BUB1B, RRM2, DLGAP5, BIRC5, KIF20A, PLK1, TOP2A, TK1, PBK, ASF1B, C18orf24, RAD54L, PTTG1, CDCA3, MCM10, PRC1, DTL, CEP55, RAD51, CENPM, CDCA8, ORC6L
KEGG hsa3030	DNA_replication	DNA2, FEN1, LIG1, MCM2, MCM3, MCM4, MCM5, MCM6, MCM7, PCNA, POLA1, POLA2, POLD1, POLD2, POLD3, POLD4, POLE, POLE2, POLE3, POLE4, PRIM1, PRIM2, RFC1, RFC2, RFC3, RFC4, RFC5, RNASEH1, RNASEH2A, RNASEH2B, RNASEH2C, RPA1, RPA2, RPA3, RPA4, SSBP1
PMID: 34019806	Tumor_Proliferation_Rate	MKI67, ESCO2, CETN3, CDK2, CCND1, CCNE1, AURKA, AURKB, E2F1, MYBL2, BUB1, PLK1, CCNB1, MCM2, MCM6
PMID: 24520177	EMT1	CLDN3, CLDN7, CLDN4, CDH1, VIM, TWIST1, ZEB1, ZEB2
PMID: 26997480	EMT2	AXL, FAP, LOXL2, ROR2, TAGLN, TWIST2, WNT5A
PMID: 34019806	EMT_signature	SNAI1, SNAI2, TWIST1, TWIST2, ZEB1, ZEB2, CDH2
PMID: 30842092	TGFβ/EMT pathway	VIM, ACTA2, COL4A1, TGFBR2, ZEB1, CLDN3, SMAD9, TWIST1, TGRB1
PMID: 29443960	Pan-F-TBRS	ACTA2, ACTG2, ADAM12, ADAM19, CNN1, COL4A1, CTGF, CTPS1, FAM101B, FSTL3, HSPB1, IGFBP3, PXDC1, SEMA7A, SH3PXD2A, TAGLN, TGFBI, TNS1, TPM1

PMID: 22553347	Angiogenesis	CDH5, SOX17, SOX18,TEK
PMID: 34019806	Endothelium	NOS3, KDR, FLT1, VCAM1, VWF, CDH5, MMRN1, ENG, CLEC14A, MMRN2
PMID: 34019806	Cancer_associated_fibroblasts	COL1A1, COL1A2, COL5A1, ACTA2, FGF2, FAP, LRP1, CD248, COL6A1, COL6A2, COL6A3, CXCL12, FBLN1, LUM, MFAP5, MMP3, MMP2, PDGFRB, PDGFRA
PMID: 34019806	Matrix	FN1, COL1A1, COL1A2, COL4A1, COL3A1, VTN, LGALS7, LGALS9, LAMA3, LAMB3, LAMC2, TNC, ELN, COL5A1, COL11A1
PMID: 30842092	DNA_damage_repair	ALKBH2, ALKBH3, APEX1, APEX2, APLF, ATM, ATR, ATRIP, BLM, BRCA1, BRCA2, BRIP1, CCNH, CDK7, CETN2, CHAF1A, CHEK1, CHEK2, CLK2, DCLRE1C, DDB1, DDB2, DUT, ENDOV, ERCC1, ERCC2, ERCC3, ERCC4, ERCC5, ERCC6, ERCC8, FAN1, FANCA, FANCB, FANCC, FANCD2, FANCE, FANCF, FANCG, FANCL, FANCM, GTF2H1, GTF2H2, GTF2H3, GTF2H4, GTF2H5, H2AFX, HLTF, HUS1, LIG1, LIG3, LIG4, MBD4, MDC1, MGMT, MLH1, MLH3, MMS19, MNAT1, MPG, MSH2, MSH3, MSH4, MSH5, MSH6, MUTYH, NEIL1, NEIL2, NEIL3, NHEJ1, NTHL1, NUDT1, OGG1, PALB2, PARP1, PARP2, PARP3, PCNA, PER1, PMS1, PMS2, PNKP, POLB, POLD1, POLE, POLG, POLH, POLL, POLM, POLQ, PRKDC, RAD1, RAD17, RAD18, RAD23A, RAD23B, RAD51C, RAD9A, RECQL4, RECQL5, RIF1, RNF168, RNF4, RNF8, RPA1, RPA2, RPA3, RPA4, RRM2B, SETMAR, SHPRH, SMUG1, TDP1, TDP2, TOPBP1, TP53, TREX1, UBE2A, UBE2B, UBE2N, UBE2V2, UNG, UVSSA, WRN, XAB2, XPA, XPC, XRCC1, XRCC4, XRCC5, XRCC6
