# Supplementary material for: Cuproptosis and Immune-Related Gene Signature Predicts Immunotherapy Response and Prognosis in Lung Adenocarcinoma
Source: Life (Basel). 2023 Jul 19;13(7):1583. doi: 10.3390/life13071583 (PMC10381686; doi:10.3390/life13071583)
Supplement: Supplementary file 1 [file life-13-01583-s001.zip › Supplementary Table 3.pdf]

Table S3. List of gene sets including 14 biological processes.

Source	Gene Signature	associated genes
KEGG has04650	Natural killer cell mediated cytotoxicity	HLA-A, HLA-B, HLA-C, HLA-G, HLA-E, KIR3DL2, KIR3DL1, KIR3DL3, KIR2DL2, KIR2DL1, KIR2DL3, KIR2DL5A, KLRC1, KLRD1, PTPN6, PTPN11, ICAM1, ICAM2, ITGAL, ITGB2, PTK2B, VAV3, VAV1, VAV2, RAC1, RAC2, RAC3, PAK1, MAP2K1, MAP2K2, MAPK1, MAPK3, TNF, CSF2, IFNG, KIR2DS1, KIR2DS3, KIR2DS4, KIR2DS5, KIR2DS2, KLRC2, KLRC3, NCR2, TYROBP, LCK, IGH, FCGR3A, FCGR3B, NCR1, NCR3, FCER1G, CD247, ZAP70, SYK, LCP2, LAT, PLCG1, PLCG2, SH3BP2, PIK3CA, PIK3CD, PIK3CB, PIK3R1, PIK3R2, PIK3R3, FYN, SHC1, SHC2, SHC3, SHC4, GRB2, SOS1, SOS2, HRAS, KRAS, NRAS, ARAF, BRAF, RAF1, MICB, MICA, ULBP1, ULBP2, ULBP3, KLRK1, KLRC4-KLRK1, HCST, CD48, CD244, PPP3CA, PPP3CB, PPP3CC, PPP3R1, PPP3R2, NFATC1, NFATC2, PRKCA, PRKCB, PRKCG, SH2D1B, SH2D1A, IFNGR1, IFNGR2, IFNA1, IFNA2, IFNA4, IFNA5, IFNA6, IFNA7, IFNA8, IFNA10, IFNA13, IFNA14, IFNA16, IFNA17, IFNA21, IFNB1, IFNAR1, IFNAR2, TNFSF10, TNFRSF10A, TNFRSF10B, FASLG, FAS, GZMB, PRF1, CASP3, BID, RAET1G, RAET1L, RAET1E
KEGG hsa04612	Antigen processing and presentation	IFNG, TNF, PSME1, PSME2, PSME3, HSPA8, HSPA1A, HSPA2, HSPA1L, HSPA1B, HSPA6, HSPA4, HSP90AA1, HSP90AB1, HLA-A, HLA-B, HLA-C, HLA-F, HLA-G, HLA-E, HSPA5, CANX, B2M, PDIA3, CALR, TAPBP, TAP1, TAP2, CD8A, CD8B, CD8B2, KIR3DL2, KIR3DL1, KIR3DL3, KIR2DL2, KIR2DL1, KIR2DL3, KIR2DL4, KIR2DL5A, KLRC1, KLRC2, KLRC3, KLRC4, KLRD1, KIR2DS1, KIR2DS3, KIR2DS4, KIR2DS5, KIR2DS2, IFI30, LGMN, CTSB, HLA-DMA, HLA-DMB, HLA-DOA, HLA-DOB, HLA-DPA1, HLA-DPB1, HLA-DQA1, HLA-DQA2, HLA-DQB1, HLA-DRA, HLA-DRB1, HLA-DRB3, HLA-DRB4, HLA-DRB5, CD74, CTSL, CTSS, CD4, CIITA, RFX5, RFXANK, RFXAP, CREB1, NFYA, NFYB, NFYC
PMID: 17624944	T cell receptor signaling	CD3D, CD3E, CD3G, CD247, CD4, CD8A, CD8B, CD8B2, PTPRC, LCK, FYN, ZAP70, LCP2, LAT, ITK, TEC, NCK1, NCK2, VAV3, VAV1, VAV2, GRAP2, GRB2, PAK1, PAK2, PAK3, PAK4, PAK5, PAK6, BUB1B-PAK6, RHOA, CDC42, DLG1, MAPK11, MAPK12, MAPK13, MAPK14, PLCG1, PPP3CA, PPP3CB, PPP3CC, PPP3R1, PPP3R2, NFATC1, NFATC2, NFATC3, SOS1, SOS2, RASGRP1, HRAS, KRAS, NRAS, RAF1, MAP2K1, MAP2K2, MAPK1, MAPK3, FOS, JUN, PRKCQ, CARD11, BCL10, MALT1, MAP3K7, MAP2K7, MAPK8, MAPK10, MAPK9, CHUK, IKBKB, IKBKG, NFKB1, RELA, NFKBIA, NFKBIB, NFKBIE, CD28, ICOS, CD40LG, PIK3R1, PIK3R2, PIK3R3, PIK3CA, PIK3CD, PIK3CB, PDPK1, AKT1, AKT2, AKT3, MAP3K8, MAP3K14, GSK3B, PDCD1, CTLA4, PTPN6, CBLB, IL2, IL4, IL5, IL10, IFNG, CSF2, TNF, CDK4
KEGG hsa04666	Fc gamma R-mediated phagocytosis	IGH, FCGR1A, FCGR2A, FCGR3A, FCGR3B, PTPRC, HCK, LYN, SYK, PIK3CA, PIK3CD, PIK3CB, PIK3R1, PIK3R2, PIK3R3, AKT1, AKT2, AKT3, RPS6KB1, RPS6KB2, PLCG1, PLCG2, PRKCD, PRKCE, RAF1, MAP2K1, MAPK1, MAPK3, PLA2G4E, PLA2G4A, JMJD7-PLA2G4B, PLA2G4B, PLA2G4C, PLA2G4D, PLA2G4F, PLA2G6, MARCKS, MARCKSL1, PLD1, PLD2, PLPP1, PLPP3, PLPP2, SPHK1, SPHK2, PRKCA, PRKCB, PRKCG, NCF1, GSN, SCIN, VAV3, VAV1, VAV2, CDC42, WAS, VASP, ARPC5, ARPC5L, ARPC4, ARPC3, ARPC1B, ARPC1A, ARPC2, RAC1, RAC2, WASF1, WASF2, WASF3, PAK1, LIMK1, LIMK2, CFL1, CFL2, PIP5K1C, PIP5K1A, PIP5K1B, ARF6, CRK, CRKL, DOCK1, ASAP1, ASAP3, ASAP2, FCGR2B, INPP5D, INPPL1, GAB2, LAT, DNM2, AMPH, BIN1, MYO10
KEGG hsa04512	ECM-receptor interaction	COL1A1, COL1A2, COL2A1, COL4A2, COL4A4, COL4A6, COL4A1, COL4A5, COL4A3, COL6A1, COL6A2, COL6A3, COL6A6, COL6A5, COL9A1, COL9A2, COL9A3, LAMA1, LAMA2, LAMA3, LAMA5, LAMA4, LAMB1, LAMB2, LAMB3, LAMB4, LAMC1, LAMC2, LAMC3, CHAD, RELN, THBS1, COMP, THBS2, THBS3, THBS4, FN1, SPP1, VTN, TNC, TNN, TNR, TNXB, NPNT, FRAS1, FREM2, FREM1, DSPP, VWF, IBSP, DMP1, AGRN, HSPG2, ITGA1, ITGA2, ITGA2B, ITGA3, ITGA4, ITGA5, ITGA6, ITGA7, ITGA8, ITGA9, ITGA10, ITGA11, ITGAV, ITGB1, ITGB3, ITGB4, ITGB5, ITGB6, ITGB7, ITGB8, CD44, SDC1, SDC4, SV2C, SV2B, SV2A, CD36, GP5, GP1BA, GP1BB, GP9, GP6, DAG1, CD47, HMMR

KEGG hsa04510	Focal adhesion	COL1A1, COL1A2, COL2A1, COL4A2, COL4A4, COL4A6, COL4A1, COL4A5, COL4A3, COL6A1, COL6A2, COL6A3, COL6A6, COL6A5, COL9A1, COL9A2, COL9A3, LAMA1, LAMA2, LAMA3, LAMA5, LAMA4, LAMB1, LAMB2, LAMB3, LAMB4, LAMC1, LAMC2, LAMC3, CHAD, RELN, THBS1, COMP, THBS2, THBS3, THBS4, FN1, SPP1, VTN, TNC, TNN, TNR, TNXB, VWF, IBSP, ITGA1, ITGA2, ITGA2B, ITGA3, ITGA4, ITGA5, ITGA6, ITGA7, ITGA8, ITGA9, ITGA10, ITGA11, ITGAV, ITGB1, ITGB3, ITGB4, ITGB5, ITGB6, ITGB7, ITGB8, PDGFA, PDGFB, PDGFC, PDGFD, EGF, IGF1, VEGFA, VEGFB, PGF, VEGFC, VEGFD, HGF, PDGFRA, PDGFRB, IGF1R, KDR, EGFR, FLT1, FLT4, MET, ERBB2, SRC, ARHGAP35, ARHGAP5, RHOA, DIAPH1, ROCK1, ROCK2, MYL2, MYL5, MYL7, MYL9, MYL10, MYL12B, MYL12A, MYLPF, PPP1CA, PPP1CB, PPP1CC, PPP1R12A, PPP1R12B, PPP1R12C, MYLK, MYLK2, MYLK3, MYLK4, PIP5K1C, PIP5K1A, PIP5K1B, ACTG1, ACTB, RASGRF1, CAPN2, ACTN1, ACTN4, TLN1, TLN2, FLNA, FLNC, FLNB, PXN, ILK, ZYX, VASP, VCL, PARVB, PARVA, PARVG, PDPK1, AKT1, AKT2, AKT3, GSK3B, CTNNB1, PRKCA, PRKCB, PRKCG, PTK2, PIK3CA, PIK3CD, PIK3CB, PIK3R1, PIK3R2, PIK3R3, PTEN, VAV3, VAV1, VAV2, RAC1, RAC2, RAC3, PAK1, PAK2, PAK3, PAK4, PAK5, PAK6, BUB1B-PAK6, CDC42, BCAR1, CRK, CRKL, DOCK1, RAPGEF1, RAP1A, RAP1B, MAPK8, MAPK10, MAPK9, JUN, BRAF, CAV1, CAV2, CAV3, FYN, SHC1, SHC2, SHC3, SHC4, GRB2, SOS1, SOS2, HRAS, RAF1, MAP2K1, MAPK1, MAPK3, ELK1, CCND1, CCND2, CCND3, BIRC2, BIRC3, XIAP, BAD, BCL2
KEGG hsa04530	Tight junction	CRB3, CLDN4, CLDN3, CLDN7, CLDN19, CLDN16, CLDN14, CLDN15, CLDN17, CLDN20, CLDN11, CLDN18, CLDN22, CLDN5, CLDN10, CLDN8, CLDN6, CLDN2, CLDN1, CLDN9, CLDN23, CLDN34, CLDN25, CLDN24, OCLN, F11R, JAM2, JAM3, BVES, CDC42, PARD6A, PARD6G, PARD6B, MPP5, MPP4, TJP3, PATJ, MPDZ, PRKCZ, PRKCI, AMOT, AMOTL1, AMOTL2, ARHGAP17, RAC1, NF2, LLGL2, LLGL1, DLG1, SCRIB, PPP2CA, PPP2CB, PPP2R1B, PPP2R1A, PPP2R2A, PPP2R2B, PPP2R2C, PPP2R2D, PARD3, TIAM1, TJP1, TJAP1, DLG2, DLG3, NEDD4, NEDD4L, CGN, CGNL1, ARHGEF2, RHOA, GATA4, MARVELD3, MAP3K1, MAPK8, MAPK10, MAPK9, JUN, CD1A, CD1B, CD1C, CD1D, CD1E, CFTR, CDK4, YBX3, SYMPK, PCNA, CCND1, ERBB2, RUNX1, HSPA4, SLC9A3R1, EZR, RDX, MSN, PRKCE, ACTG1, ACTB, CACNA1D, MAP3K5, MAP2K7, SRC, CTTN, HCLS1, ACTR2, ACTR3B, ACTR3C, ACTR3, WHAMM, WAS, VASP, PRKACA, PRKACB, PRKACG, RAB13, ARHGEF18, ROCK1, ROCK2, MYL2, EPB41L4B, STK11, PRKAA1, PRKAA2, PRKAB1, PRKAB2, PRKAG1, PRKAG3, PRKAG2, MYH9, MYH10, MYH11, MYH14, MYL6B, MYL6, MYL9, MYL12B, MYL12A, IGSF5, MAGI1, SYNPO, ACTN1, ACTN4, MICALL2, RAB8A, RAB8B, RAPGEF6, RAP1A, ITGB1, AFDN, TJP2, RAPGEF2, RAP2C, MARVELD2, TUBA1B, TUBA4A, TUBA3C, TUBA1A, TUBA1C, TUBA8, TUBA3E, TUBA3D, TUBAL3
KEGG hsa04115	p53 signaling	ATM, CHEK2, ATR, CHEK1, GORAB, CDKN2A, MDM2, MDM4, TP53, CDKN1A, CCND1, CCND2, CCND3, CDK4, CDK6, CCNE1, CCNE2, CDK2, SFN, RPRM, CCNB1, CCNB2, CDK1, GADD45A, GADD45B, GADD45G, GTSE1, FAS, PIDD1, TNFRSF10A, TNFRSF10B, CASP8, BID, BAX, PMAIP1, BBC3, TP53AIP1, SIVA1, BCL2L1, BCL2, TP53I3, EI24, SHISA5, PERP, ZMAT3, SIAH1, CYCS, APAF1, CASP9, CASP3, AIFM2, IGFBP3, IGF1, SERPINE1, ADGRB1, CD82, THBS1, SERPINB5, DDB2, RRM2B, RRM2, SESN1, SESN3, SESN2, PTEN, TSC2, STEAP3, COP1, RCHY1, CCNG1, CCNG2, PPM1D, TP73
KEGG hsa3430	Mismatch repair	SSBP1, PMS2, MLH1, MSH6, MSH2, MSH3, MLH3, RFC1, RFC4, RFC2, RFC5, RFC3, PCNA, EXO1, RPA1, RPA2, RPA3, RPA4, POLD1, POLD2, POLD3, POLD4, LIG1
KEGG hsa3440	Homologous recombination	SSBP1, RAD50, MRE11, NBN, ATM, BRCA1, BARD1, RBBP8, BRIP1, TOPBP1, ABRAXAS1, UIMC1, BABAM1, BABAM2, BRCC3, PALB2, BRCA2, SEM1, SYCP3, RPA1, RPA2, RPA3, RPA4, RAD51, RAD52, RAD51B, RAD51C, RAD51D, XRCC2, XRCC3, RAD54L, RAD54B, POLD1, POLD2, POLD3, POLD4, BLM, TOP3A, TOP3B, MUS81, EME1

KEGG ko04151	PI3K-Akt signaling	EGF, TGFA, EREG, AREG, FGF1, FGF2, FGF3, FGF4, FGF17, FGF6, FGF7, FGF8, FGF9, FGF10, FGF16, FGF5, FGF18, FGF20, FGF22, FGF19, FGF21, FGF23, NGF, BDNF, NTF3, NTF4, INS, IGF1, IGF2, PDGFA, PDGFB, PDGFC, PDGFD, CSF1, KITLG, FLT3LG, VEGFA, VEGFB, PGF, VEGFC, VEGFD, HGF, ANGPT1, ANGPT2, ANGPT4, EFNA1, EFNA2, EFNA3, EFNA4, EFNA5, EGFR, ERBB2, ERBB3, ERBB4, FGFR1, FGFR2, FGFR3, FGFR4, NGFR, NTRK1, NTRK2, INSR, IGF1R, PDGFRA, PDGFRB, CSF1R, KIT, FLT3, FLT1, FLT4, KDR, MET, TEK, EPHA2, GRB2, SOS1, SOS2, HRAS, KRAS, NRAS, RAF1, MAP2K1, MAP2K2, MAPK1, MAPK3, IRS1, TLR2, TLR4, RAC1, IGH, SYK, CD19, PIK3AP1, GH1, GH2, CSH1, CSH2, PRL, OSM, IL2, IL3, IL6, IL4, IL7, IFNA1, IFNA2, IFNA4, IFNA5, IFNA6, IFNA7, IFNA8, IFNA10, IFNA13, IFNA14, IFNA16, IFNA17, IFNA21, IFNB1, EPO, CSF3, GHR, PRLR, OSMR, IL2RA, IL2RB, IL2RG, IL3RA, IL6R, IL4R, IL7R, IFNAR1, IFNAR2, EPOR, CSF3R, JAK1, JAK2, JAK3, COL1A1, COL1A2, COL2A1, COL4A2, COL4A4, COL4A6, COL4A1, COL4A5, COL4A3, COL6A1, COL6A2, COL6A3, COL6A6, COL6A5, COL9A1, COL9A2, COL9A3, LAMA1, LAMA2, LAMA3, LAMA5, LAMA4, LAMB1, LAMB2, LAMB3, LAMB4, LAMC1, LAMC2, LAMC3, CHAD, RELN, THBS1, COMP, THBS2, THBS3, THBS4, FN1, SPP1, VTN, TNC, TNN, TNR, TNXB, VWF, IBSP, ITGA1, ITGA2, ITGA2B, ITGA3, ITGA4, ITGA5, ITGA6, ITGA7, ITGA8, ITGA9, ITGA10, ITGA11, ITGAV, ITGB1, ITGB3, ITGB4, ITGB5, ITGB6, ITGB7, ITGB8, PTK2, PIK3CA, PIK3CD, PIK3CB, PIK3R1, PIK3R2, PIK3R3, F2R, CHRM1, CHRM2, LPAR1, LPAR2, LPAR3, LPAR4, LPAR5, LPAR6, GNB1, GNB2, GNB3, GNB4, GNB5, GNG2, GNG3, GNG4, GNG5, GNG7, GNG8, GNG10, GNG11, GNG12, GNG13, GNGT1, GNGT2, PIK3CG, PIK3R5, PIK3R6, PDPK1, STK11, PRKAA1, PRKAA2, DDIT4, TSC1, TSC2, RHEB, MLST8, MTOR, RPTOR, EIF4EBP1, EIF4E, EIF4E2, EIF4E1B, RPS6KB1, RPS6KB2, EIF4B, RPS6, PRKCA, PKN1, PKN2, PKN3, SGK1, SGK2, SGK3, C8orf44-SGK3, AKT1, AKT2, AKT3, MAGI1, MAGI2, PTEN, THEM4, PPP2CA, PPP2CB, PPP2R1B, PPP2R1A, PPP2R2A, PPP2R2B, PPP2R2C, PPP2R2D, PPP2R3B, PPP2R3C, PPP2R3A, PPP2R5B, PPP2R5C, PPP2R5D, PPP2R5E, PPP2R5A, HSP90AA1, HSP90AB1, HSP90B1, CDC37, CRTC2, PHLPP1, PHLPP2, TCL1A, TCL1B, MTCP1, NOS3, BRCA1, GSK3B, GYS2, GYS1, PCK1, PCK2, G6PC, G6PC2, G6PC3, MYC, CCND1, CDKN1A, CDKN1B, CDK2, CDK4, CDK6, CCND2, CCND3, CCNE1, CCNE2, FOXO3, RBL2, FASLG, BCL2L11, YWHAZ, YWHAB, YWHAQ, YWHAE, YWHAH, YWHAG, BAD, BCL2L1, BCL2, CASP9, CREB1, ATF2, ATF4, CREB3, CREB3L1, CREB3L2, CREB3L3, CREB3L4, CREB5, ATF6B, MCL1, RXRA, NR4A1, IKBKG, CHUK, IKBKB, RELA, NFKB1, MYB, MDM2, TP53
PMID: 25970248	Wnt signaling	PORCN, WNT1, WNT2, WNT2B, WNT3, WNT3A, WNT4, WNT5A, WNT5B, WNT6, WNT7A, WNT7B, WNT8A, WNT8B, WNT9A, WNT9B, WNT10B, WNT10A, WNT11, WNT16, CER1, NOTUM, WIF1, SERPINF1, SOST, DKK1, DKK2, DKK4, SFRP1, SFRP2, SFRP4, SFRP5, RSPO1, RSPO2, RSPO3, RSPO4, LGR4, LGR5, LGR6, RNF43, ZNRF3, FZD1, FZD7, FZD2, FZD3, FZD4, FZD5, FZD8, FZD6, FZD10, FZD9, LRP5, LRP6, BAMBI, CSNK1E, TPTEP2-CSNK1E, DVL3, DVL2, DVL1, FRAT1, FRAT2, CSNK2A1, CSNK2A2, CSNK2A3, CSNK2B, NKD1, NKD2, CXXC4, SENP2, GSK3B, CTNNB1, AXIN1, AXIN2, APC, APC2, CSNK1A1L, CSNK1A1, TCF7, TCF7L1, TCF7L2, LEF1, CTNNBIP1, CBY1, CHD8, SOX17, CTBP1, CTBP2, CTNND2, CREBBP, EP300, RUVBL1, SMAD4, SMAD3, MAP3K7, NLK, MYC, JUN, FOSL1, CCND1, CCND2, CCND3, CCN4, PPARD, MMP7, PSEN1, PRKACA, PRKACB, PRKACG, TP53, SIAH1, CACYBP, SKP1, TBL1X, TBL1Y, TBL1XR1, BTRC, FBXW11, CUL1, RBX1, GPC4, ROR1, ROR2, RYK, VANGL2, VANGL1, PRICKLE1, PRICKLE2, PRICKLE4, PRICKLE3, INVS, DAAM1, DAAM2, RHOA, ROCK2, RAC1, RAC2, RAC3, MAPK8, MAPK10, MAPK9, PLCB1, PLCB2, PLCB3, PLCB4, CAMK2A, CAMK2D, CAMK2B, CAMK2G, PPP3CA, PPP3CB, PPP3CC, PPP3R1, PPP3R2, PRKCA, PRKCB, PRKCG, NFATC1, NFATC2, NFATC3, NFATC4
PMID: 20495575	TGF-beta signaling	CHRD, NOG, NBL1, MICOS10-NBL1, GREM1, GREM2, THBS1, DCN, FMOD, LEFTY1, LEFTY2, FST, BMP2, BMP4, BMP6, INHBB, BMP5, BMP7, BMP8B, BMP8A, GDF5, GDF6, GDF7, AMH, THSD4, FBN1, LTBP1, TGFB1, TGFB2, TGFB3, INHBA, INHBC, INHBE, NODAL, NEO1, HJV, BMPR1A, BMPR1B, ACVR1, BMPR2, ACVR2A, RGMA, RGMB, AMHR2, TGFBR1, TGFBR2, ACVR1B, ACVR2B, ACVR1C, BAMBI, SMAD1, SMAD5, SMAD9, SMAD2, SMAD3, SMAD4, SMAD6, SMAD7, SMURF1, SMURF2, ZFYVE9, ZFYVE16, HAMP, ID1, ID2, ID3, ID4, RBL1, E2F4, E2F5, TFDP1, CREBBP, EP300, SP1, TGIF1, TGIF2, MYC, CDKN2B, PITX2, RBX1, CUL1, SKP1, MAPK1, MAPK3, IFNG, TNF, RHOA, ROCK1, PPP2R1B, PPP2R1A, PPP2CA, PPP2CB, RPS6KB1, RPS6KB2

KEGG hsa4110	Cell cycle	CCND1, CCND2, CCND3, CDK4, CDK6, RB1, RBL1, RBL2, ABL1, HDAC1, HDAC2, E2F1, E2F2, E2F3, E2F4, E2F5, TFDP1, TFDP2, GSK3B, TGFB1, TGFB2, TGFB3, SMAD2, SMAD3, SMAD4, MYC, ZBTB17, CDKN2A, CDKN2B, CDKN2C, CDKN2D, CDKN1B, CDKN1C, CDKN1A, CCNE1, CCNE2, CDK2, SKP1, CUL1, RBX1, SKP2, CCNA2, CCNA1, CDC6, CDC45, CDC7, DBF4, CDK1, CCNB1, CCNB2, CCNB3, CDC25B, CDC25C, YWHAZ, YWHAB, YWHAQ, YWHAE, YWHAH, YWHAG, PLK1, WEE1, WEE2, PKMYT1, CCNH, CDK7, ANAPC1, ANAPC2, CDC27, ANAPC4, ANAPC5, CDC16, ANAPC7, CDC23, ANAPC10, ANAPC11, CDC26, ANAPC13, CDC20, PTTG1, PTTG2, ESPL1, SMC1A, SMC1B, SMC3, STAG2, STAG1, RAD21, TTK, BUB1, BUB3, BUB1B, MAD1L1, MAD2L1, MAD2L2, FZR1, CDC14B, CDC14A, ATR, ATM, TP53, CHEK1, CHEK2, CREBBP, EP300, PRKDC, MDM2, GADD45A, GADD45B, GADD45G, PCNA, SFN, CDC25A, ORC1, ORC2, ORC3, ORC4, ORC5, ORC6, MCM2, MCM3, MCM4, MCM5, MCM6, MCM7
