# Supplementary material for: Cuproptosis and Immune-Related Gene Signature Predicts Immunotherapy Response and Prognosis in Lung Adenocarcinoma
Source: Life (Basel). 2023 Jul 19;13(7):1583. doi: 10.3390/life13071583 (PMC10381686; doi:10.3390/life13071583)
Supplement: Supplementary file 1 [file life-13-01583-s001.zip › Supplementary Table 4.pdf]

Table S4. Univariate and multivariate Cox regression analyses for risk stratification.

Variables	Low-risk subgroup		High-risk subgroup	
	HR (95% CI)	*P* value	HR (95% CI)	*P* value
Univariate Cox regression				
Age	1.02 (1-1.05)	0.096	1.01 (0.99-1.03)	0.179
Gender	0.98 (0.61-1.57)	0.924	0.99 (0.67-1.46)	0.974
Stage	2.1 (1.23-3.59)	0.007	2.3 (1.55-3.41)	<0.001
TMB	0.97 (0.93-1.02)	0.276	0.99 (0.95-1.02)	0.371
RiskScore	1.75 (0.46-6.74)	0.413	1.64 (1.09-2.46)	0.017
Multivariate Cox regression				
Age	1.02 (0.99-1.05)	0.216	1.01 (0.99-1.03)	0.17
Gender	0.93 (0.57-1.52)	0.766	0.94 (0.63-1.38)	0.737
Stage	1.97 (1.08-3.56)	0.026	2.33 (1.56-3.47)	<0.001
TMB	0.98 (0.93-1.03)	0.362	0.99 (0.95-1.03)	0.569
RiskScore	1.34 (0.3-6.07)	0.703	1.74 (1.15-2.65)	0.009
